# Cost-Efficient High-Resolution Linear Absorption Spectra through Extrapolating the Dipole Moment from Real-Time Time-Dependent Electronic-Structure Theory

**DOI:** 10.1021/acs.jctc.3c00727

**Published:** 2023-10-24

**Authors:** Eirill Hauge, Håkon Emil Kristiansen, Lukas Konecny, Marius Kadek, Michal Repisky, Thomas Bondo Pedersen

**Affiliations:** † Hylleraas Centre for Quantum Molecular Sciences, Department of Chemistry, University of Oslo, P.O. Box 1033, Blindern, 0315 Oslo, Norway; ‡ Department of Numerical Analysis and Scientific Computing, Simula Research Laboratory, Kristian Augusts Gate 23, 0164 Oslo, Norway; ¶ Hylleraas Centre for Quantum Molecular Sciences, Department of Chemistry, University of Tromsø—The Arctic University of Norway, N-9037 Tromsø, Norway; § Center for Free Electron Laser, Max Planck Institute for the Structure and Dynamics of Matter Science, Luruper Chaussee 149, 22761 Hamburg, Germany; ∥ Department of Physics, Northeastern University, Boston, Massachusetts 02115, United States; ⊥ Department of Physical and Theoretical Chemistry, Faculty of Natural Sciences, Comenius University, Ilkovicova 6, SK-84215 Bratislava, Slovakia

## Abstract

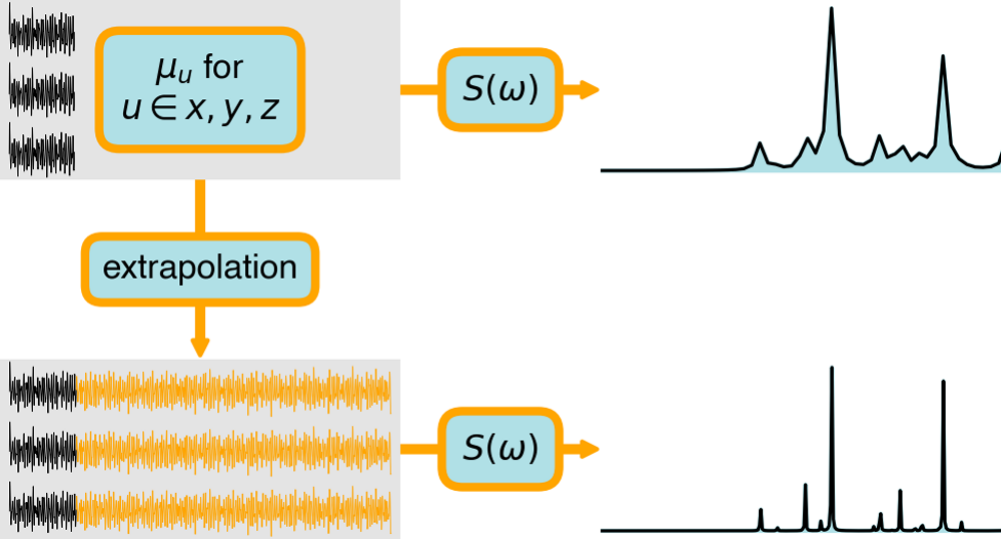

We present a novel function fitting method for approximating the propagation of the time-dependent electric dipole moment from real-time electronic structure calculations. Real-time calculations of the electronic absorption spectrum require discrete Fourier transforms of the electric dipole moment. The spectral resolution is determined by the total propagation time, i.e., the trajectory length of the dipole moment, causing a high computational cost. Our developed method uses function fitting on shorter trajectories of the dipole moment, achieving arbitrary spectral resolution through extrapolation. Numerical testing shows that the fitting method can reproduce high-resolution spectra by using short dipole trajectories. The method converges with as little as 100 a.u. dipole trajectories for some systems, though the difficulty converging increases with the spectral density. We also introduce an error estimate of the fit, reliably assessing its convergence and hence the quality of the approximated spectrum.

## 1 Introduction

The rapid advancement of laser technology in the past decades allows us to probe matter on spatiotemporal scales that approach the characteristic time and length scales of the electron, opening the field of attosecond science.^1,2^ This development has forced quantum chemists to shift their attention from the time-independent to the time-dependent Schrödinger and Dirac equations.^3−5^ Numerical approaches to laser-driven electron dynamics are often labeled *real-time* methods to distinguish them from the response-theoretical methods to the time-dependent Schrödinger/Dirac equation, the latter solving the equations of motion perturbatively in the frequency domain.^6,7^

Even without explicit reference to results derived from perturbation theory such as, e.g., Fermi’s golden rule, it is still possible to extract linear and low-order nonlinear optical properties from nonperturbative real-time simulations, including electronic absorption spectra and time-resolved pump–probe absorption spectra that would be hard or impossible to compute using response theory—see refs (8–12) for recent examples.

In this work, we focus on electronic absorption spectra extracted from electron-dynamics simulations driven by a Dirac-delta impulse, which excites the molecule into all dipole-allowed excited states simultaneously.^8^ Due to the nonperturbative nature of real-time methods, the resulting spectrum contains nonlinear (e.g., two-photon) as well as linear absorption lines.^11^ For weak pulses, the nonlinear effects are small, and the absorption spectrum is dominated by linear lines. In practice, the induced electric dipole moment is recorded in the course of the simulation and subsequently transformed to the frequency domain to yield an absorption cross section. When using the conventional discrete Fourier transform to process the signal, the spectral resolution is inversely proportional to the number of time steps *N* and the time-step length *Δt*, as *Δω* = 2π/(*NΔt*). Obtaining sufficient spectral resolution typically requires tens to hundreds of thousands of time steps since *Δt* cannot be increased beyond a certain limit if rapid oscillations of the electron density are to be captured. Moreover, increasing *Δt* reduces the accuracy and stability of the numerical integration scheme used to propagate the electronic degrees of freedom. As the computational effort in each time step requires multiple rebuilds of the Hamiltonian matrix, it is comparable to several iterations of a ground-state optimization within the chosen electronic-structure model.^13^ Hence, there is considerable interest in decreasing the number of time steps required to achieve sufficient spectral resolution.

In addition to reducing the number of time steps, it is possible to increase the computational efficiency of real-time electronic structure methods by disregarding negligible basis functions,^14^ basis-function pairs and quartets.^15^ As a result, in a large molecule, although there are  electron repulsion integrals (ERIs) in total, it can be shown that only  of them are significant, where *N* refers to the number of basis functions. As shown within real-time time-dependent density-functional theory (RT-TDDFT), a large prefactor associated with the evaluation of non-negligible ERIs can further be reduced by using, e.g., the resolution-of-the-identity method.^16^ By applying a spatial truncation radius upon the time-dependent density matrix, RT-TDDFT can approach the linear  scaling.^17,18^

Previous efforts to improve the spectral resolution have been made by estimating excitation energies through various signal processing techniques.^19−21^ More recently, Bruner et al.^22^ investigated the use of Padé approximants to interpolate the discrete Fourier transforms used for the absorption spectrum. These are all methods operating in the frequency domain, leaving no other validation options other than comparison with a fully propagated spectrum.

The original periodic signal is typically damped using a decaying exponential function to reduce unwanted artifacts arising when the discrete Fourier transform is applied to oscillating functions in simulations with finite trajectory length. In the time domain, the number of time points can be increased by padding the damped signal with zeros, leading to finer spectra. However, this artificial extension of the trajectory length can be applied only to sufficiently damped signals.

In this work, we investigate a more sophisticated and powerful alternative: the extrapolation of a short signal. The discrete Fourier transform of an extrapolated signal achieves an increasingly higher spectral resolution as the extrapolation length increases. This requires the development of a stable and reliable method for time-series forecasting. The inherently harmonic character of the time-dependent wave function in the absence of an external field suggests that such forecasting of molecular properties should be possible. Importantly, the forecasted signal can be verified in the time domain by comparing it with a relatively few additional time steps. To the best of our knowledge, no published work exists on improving the spectral resolution by such an extrapolation of the time-dependent dipole moment.

The current success and popularity of machine learning is undeniable, including use cases in chemistry,^23–29^ and one might be tempted to leverage artificial neural networks for forecasting the time-dependent electric-dipole moment. However, while artificial neural networks are powerful tools for pattern detection in large data sets, they struggle with precise and reliable extrapolations.^30,31^ Although the universal approximation theorem^32^ tells us that an excellent *interpolation* can be achieved, it does not guarantee a stable *extrapolation*. In order to achieve a stable extrapolation, overfitting must be avoided by enforcing sufficient restrictions.

In this article, we present a novel approach for obtaining high-resolution absorption spectra from real-time simulations of laser-driven electron dynamics by exploiting *a priori* knowledge of the form of the dipole function from quantum mechanics in a finite-dimensional Hilbert space. The form of the dipole function thus is motivated by the underlying physics, with unknown parameters to be determined by fitting a short dipole trajectory from a real-time simulation. The fitted function may be evaluated at any point in time, meaning that it can be extrapolated in the time domain to arbitrary future time. This further implies that we can achieve arbitrary spectral resolution. For sufficiently weak Dirac-delta impulse, the evaluation of absorption spectra based on these fitted functions may use analytical expressions for the linear response function.^6^

Working in the time domain, a quantitative error measure of the fitted dipole function can be monitored during the course of the real-time simulation and used to evaluate convergence. This way, an unnecessarily long real-time propagation can be avoided by automatically terminating the propagation upon convergence of the fit. The developed method is independent of the quantum mechanical model and is tested with several molecular systems using mainly RT-TDDFT.^8,33–38^ Despite certain flaws arising mainly from the almost universally adopted adiabatic density-functional approximation,^3,39^ RT-TDDFT is the far most widely used electronic-structure method for laser-driven electron dynamics. With computational costs comparable to (or *below*) time-dependent Hartree–Fock theory,^40^ RT-TDDFT takes into account electron-correlation effects that would otherwise require advanced and computationally expensive wave function theories.^4,5^ To demonstrate the independence of the underlying electronic-structure theory, we also present results obtained from real-time time-dependent configuration interaction singles (RT-TDCIS)^41−43^ theory.

We start with a short presentation of the electric-dipole approximation within real-time simulations before introducing the proposed method for fitting the time-dependent electric-dipole moment. After briefly laying out the simulation details for the real-time simulation of a selection of systems, the results of the fitting method for these systems are presented and discussed. Finally, we reflect on the performance of the fitting method and discuss potential future improvements.

## 2 Theory

In this work, we employ the following conventions: Subscripts *u*, *v* denote Cartesian components, vectors are typed in boldface, and quantum-mechanical operators are denoted by a hat. Following the convention of response theory by Olsen and Jørgensen,^6^ we define the Fourier transform and its inverse according to
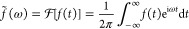
1
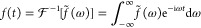
2where the transformed function is denoted by a tilde. Atomic units are used throughout, unless otherwise specified.

### 2.1 Real-Time Simulations of Absorption Spectra

Within the clamped-nucleus Born–Oppenheimer approximation, real-time simulations of electronic absorption spectra typically assume the electric-dipole approximation where a molecule is subjected to a time-dependent spatially uniform electric field, *F*(*t*). The time-dependent Hamiltonian reads
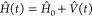
3where *Ĥ*_0_ is the time-independent electronic Hamiltonian, and the interaction operator is given by
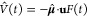
4where **μ̂** is the electric dipole moment operator. The linear polarization direction of the electric field is determined by real unit vector **u**, such that the field aligns with one of the Cartesian axes. This implies the form *V̂*(*t*) = −μ̂_*u*_*F*(*t*), where μ̂_*u*_ is the component of **μ̂** along the polarization direction.

We assume that the electronic system is in the ground state |0⟩ at time *t* < 0, and that the external field *F*(*t*) is only active between *t* = 0 and time *t*_0_ ≥ 0. At time *t*_0_, the Hamiltonian reduces to the time-independent Hamiltonian such that Schrödinger’s equation for *t* ≥ *t*_0_ becomes
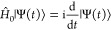
5The time-dependent wave function in the absence of the external field oscillates around the solution at time *t*_0_, |Ψ(*t*_0_)⟩ = ∑_*n*_*k*_*n*_(*t*_0_) |*n*⟩, as given by
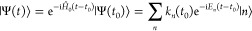
6where |*n*⟩ denotes a normalized eigenfunction of the unperturbed Hamiltonian, *Ĥ*_0_ |*n*⟩ = *E*_*n*_ |*n*⟩.^44,45^ This formulation is exact when the electronic continuum is excluded, e.g., by choosing a fixed, finite basis, as commonly done in quantum chemistry. Actual simulations are not performed in the energy eigenbasis but in, e.g., a basis of Slater determinants, implying that the coefficients *k*_*n*_(*t*_0_) are not known.

In order to obtain the electronic absorption spectrum averaged over all molecular orientations relative to the electric field, the time-dependent electric dipole moment μ_*u*_(*t*) = ⟨Ψ(*t*)|μ̂_*u*_|Ψ(*t*)⟩ is calculated in three separate simulations with the electric field polarized in each of the three Cartesian directions (*u* = *x*, *y*, *z*). The absorption cross-section is then obtained from the Fourier transform of the dipole moments, μ̃_*u*_(ω), as^46^
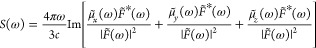
7where *c* is the speed of light. The resulting spectrum contains both linear (one-photon transitions between the ground and excited states) and nonlinear (multiphoton transitions between the ground and excited states and one- and multiphoton transitions between excited states) absorptions, as recently stressed by Guandalini et al.^11^ We note that only the *induced* dipole moment, that is, the total dipole moment with the static ground-state part subtracted, contributes to the absorption cross section, but for notational convenience, we only distinguish between that and the total dipole moment when it is strictly required.

Since the dipole moment is calculated on a finite discrete time grid, the Fourier transforms are replaced by discrete Fourier transforms, thus introducing artificial periodic boundary conditions. To avoid artifacts from these, the dipole moment is multiplied by a damping factor before the discrete Fourier transform, i.e.,
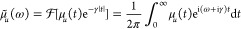
8where we have used that the induced dipole moment vanishes for *t* < 0. The Fourier transform thus becomes a Laplace transform. The parameter  can be interpreted as a common (inverse) lifetime of all excited states, giving rise to Lorentzian line shapes in the simulated absorption spectra.^47^ The discrete Fourier transform, however, requires a very large, often prohibitive, number of time steps to achieve sufficient spectral resolution. In the following sections, we describe an extrapolation technique aiming at high resolution with a short simulation time.

### 2.2 Expected Form of the Electric Dipole Moment

Once the external field is turned off, the time-dependent electric dipole moment evolves according to μ_*u*_(*t*) = ⟨Ψ(*t*)|μ̂_*u*_|Ψ(*t*)⟩, where |Ψ(*t*)⟩ is defined in eq 6. The dipole moment oscillates with the Bohr frequencies ω_*nm*_ = *E*_*n*_ – *E*_*m*_ according to
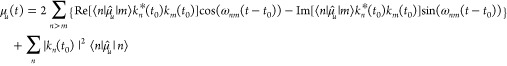
9for time *t* ≥ *t*_0_.^48^ The function form of the approximated dipole moment μ_*u*_(*t*) ≈ μ_*u*_(*t*) will therefore be given by
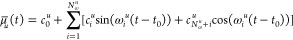
10where *N*_ω_^*u*^ is the number of participating frequencies ω_*i*_^*u*^, each frequency with two independent linear coefficients c_*i*_^*u*^ and *c*_*N*_ω_^*u*^+*i*_^*u*^. If we can determine these frequencies and their corresponding real coefficients from a short dipole time series, then we obtain a *continuous* dipole function and, hence, infinite spectral resolution.

As described in detail below, we estimate the participating frequencies using the poles of a Fourier–Padé approximant, while the linear coefficients are determined using linear regression in a subsequent step.

### 2.3 Estimating Bohr Frequencies

In order to estimate the frequencies of the dipole moment, we investigate the singular points of the Fourier–Padé approximant, originally introduced in real-time quantum simulations by Bruner et al.^22^ In general, the Padé approximant is used to accelerate convergence of a truncated power series. The discrete Fourier transform can be written as the power series
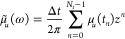
11where *z* depends on the frequency according to
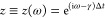
12The diagonal Fourier–Padé approximates the Fourier transform using two polynomials *P*_*u*_(*z*) and *Q*_*u*_(*z*) of degree *M* = (*N*_*t*_ – 1)/2
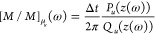
13where the coefficients of the polynomials create a Toeplitz linear system, for details see ref (22). The Fourier–Padé poles, denoted *z*_*p*_^*u*^, are found by
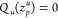
14where the damping parameter γ is set to zero, as the damping parameter removes the singularities of the spectrum. The Bohr frequencies are positive and real-valued, while the frequencies corresponding to the roots of *Q*_*u*_(*z*) will be complex. The number of roots of *Q*_*u*_(*z*), (which amounts to *M* roots), should also significantly exceed the number of Bohr frequencies. The potential frequencies are given by
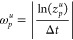
15where ln(*z*_*p*_^*u*^) returns the principal value of the logarithm. Only roots Im(*z*_*p*_^*u*^) > 0 are considered, as the complex conjugate root theorem states that complex roots will form conjugate pairs. These conjugate pairs yield duplicates of the real-valued frequencies. Real-valued roots *z*_*p*_^*u*^ yield purely imaginary frequencies and are therefore also excluded. The potential frequencies ω_*p*_^*u*^ discard the imaginary component and should represent the extrema of the Fourier–Padé spectrum, not singular points like *z*_*p*_^*u*^.

Estimating the potential frequencies uses the Python NumPy^49^ library to compute the eigenvalues of the companion matrix^50^ of the polynomial *Q*_*u*_(*z*) to determine its roots. This method exhibits poor scaling with respect to the number of time points, *N*_*t*_, representing a computational bottleneck of the dipole-moment μ_*u*_(*t*) fitting procedure. In real-time simulations using very small time steps, one may safely increase the step length on the dipole data used to create the Fourier–Padé approximant to alleviate the computational cost. As shown by Mattiat and Luber,^51^ the convergence of the Fourier–Padé approximant is mostly impacted by the trajectory length *N*_*t*_*Δt*, and not the time step itself. However, the discrete Fourier transform and hence also the Fourier–Padé are periodic with a cycle length of 2π/*Δt*. Peaks above π/*Δt* will *fold back* due to antisymmetry and appear as negative duplicates polluting the spectrum. Therefore, it is crucial to keep the time step *Δt* < π/ω_max_, where ω_max_ is the largest significant frequency in the signal.

The potential frequencies ω_*p*_^*u*^ must be classified as either an estimated frequency or a redundant root. The classification is based on the assumption that ln (*z*_*p*_^*u*^)/(i*Δt*) should have a significant imaginary component if ω_*p*_^*u*^ is a redundant root, while it should lie close to the real axis if ω_*p*_^*u*^ corresponds to an actual Bohr frequency. This further means that *Q*_*u*_(*z*(ω_*p*_^*u*^)) should be close to zero and that [*M*/*M*]_*μ_u_*_(ω_*p*_^*u*^) should be large for estimated frequencies. Hence, we create a two-dimensional representation **r**_*p*_^*u*^ of the prospective frequencies ω_*p*_^*u*^ given by
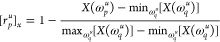
16
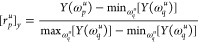
17where the unnormalized features are defined as
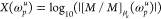
18
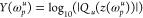
19The base-10 logarithm is used to manage the extreme scaling of both features, as prospective frequencies should cause *Q*_*u*_(*z*(ω_*p*_^*u*^)) to approach zero and hence be a nearly singular point of [*M*/*M*]_*μ_u_*_(ω_*p*_^*u*^). The features are constructed such that estimated frequencies should be close to **r**_*p*_^*u*^ = (0, 0), while redundant roots should be closer to **r**_*p*_^*u*^ = (1, 1).

We use the *K*-means clustering algorithm (see, e.g., refs (52 and 53)), implemented in the Python SciKit-Learn^54^ library, with *K* = 2 to classify prospective frequencies. The 2-means clustering algorithm is a computationally inexpensive way to separate a set into two categories. The *centroid* for the cluster of potential frequencies should be closer to (0, 0), whereas the centroid for the redundancy cluster should be closer to (1, 1).

### 2.4 Determining Linear Coefficients

Once the frequencies are estimated, the linear coefficients are determined by using linear regression. The coefficients are optimized by minimizing the cost function^55^
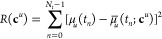
20Using the general form of the dipole moment in eq 9, the linear coefficients may be optimized using a simple least-squares optimization. The only restraint in the optimization of these coefficients is that they are real. This fitting procedure is general for any type of external field, *F*(*t*). However, as shown by Hauge,^48^ restricting the coefficients is crucial to avoid overfitting the dipole moment.

The form μ_*u*_(*t*) in eq 10 is based on the full dipole moment, correct through all orders in perturbation theory, and is independent of the electric field. In this work, we use a Dirac delta-type impulse^8^ of strength κ
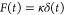
21which has an infinitely wide frequency distribution and thus generates the full absorption spectrum for the given polarization direction. This implies that *t*_0_ = 0. Further, we assume that the electric field strength is sufficiently weak, such that we may regard the interaction operator *V̂*(*t*) as a time-dependent perturbation and assume that the interaction induces only one-photon transitions from the ground state, i.e., a linear absorption spectrum. The electric dipole moment should then be of the form μ_*u*_(*t*) ≈ μ_*u*_^(0)^ + μ_*u*_^(1)^(*t*), where the zeroth order dipole moment corresponds to the ground-state value, μ_*u*_^(0)^ = μ_*u*_(*t* = 0). We now investigate an analytical expression for the first-order correction of the dipole moment induced by a weak Dirac delta impulse.

We start with the exact expression for the linear response function^6^
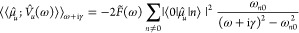
22where we have used the Fourier transform of the interaction operator *V̂*(*t*)
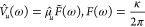
23The linear response function and the first-order correction to the dipole moment are related by . Since μ_*u*_^1^(*t* < 0) = 0, we get the relation
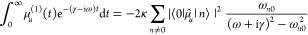
24Using well-known Laplace transforms, it is readily verified that the first-order dipole correction must be a linear combination of sine waves^48^
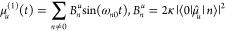
25We have also used that the first-order perturbation correction to the dipole moment should only include one-photon transitions. This further means that the approximated dipole moment, when using a weak Dirac delta impulse, should have the form
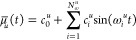
26where all sine coefficients are positive.

The coefficients of μ_*u*_(*t*), approximating the dipole moment from the Dirac delta impulse, are optimized using the *least absolute shrinkage and selection operator* (LASSO)^56^ method. The coefficients are determined according to
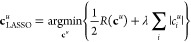
27where λ is the *shrinkage parameter* restricting the magnitude of the coefficients, *c*_*i*_^*u*^. In contrast to the ordinary linear least-squares algorithm, the LASSO method is iterative and, therefore, somewhat less computationally efficient. In return, this makes it possible to enforce positive coefficients, as in the implementation by SciKit-Learn.^54^ This makes the method less prone to overfitting.

### 2.5 Molecular Orbital Decomposition

The electric dipole moment can be written as a sum of contributions from elementary molecular orbital (MO) transitions^8^
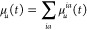
28where *i* and *a* label occupied and virtual MOs, respectively. The components μ_*u*_^*ia*^(*t*) are then approximated separately. This MO decomposition can divide a dense spectrum into a series of sparser spectra and aid in the assignment of absorption lines.^8^ Clustering the MO components into groups can be used to offset the increased memory consumption.^57^ For the fitting method, the creation of clusters with well-separated frequencies could also reduce the accumulation of errors when summing the component fits.

When the individual components are fitted, the assumptions on the sign of the linear coefficients are no longer valid. As is clear from the underlying theory and as demonstrated in practice by Bruner et al.,^22^ the same frequencies may be found in several components μ_*u*_^*ia*^, and their corresponding partial spectra may contain negative peaks. Only the full spectrum, i.e., the sum of the components, is guaranteed to contain positive peaks exclusively. The ordinary least-squares method must therefore be used when optimizing the linear coefficients of the individual components, which may introduce additional errors due to overfitting in each component.

Alternatively, the fitting algorithm may estimate the frequencies of each component separately and then optimize the linear coefficients for the full dipole moment. This way, the additional coefficient restrictions can be used in the optimization. In our experience, however, this produces a vast number of estimated frequencies leading to problems with overfitting even when enforcing positive linear coefficients.

### 2.6 Convergence Criterion

The goal of the fitting method is to accurately construct the function μ(*t*) using the shortest possible dipole trajectory. A given trajectory is divided into two parts: a fitting domain and a verification domain. The linear coefficients are optimized by using only the fitting domain, while the error is calculated on the verification domain. When estimating the frequencies, however, the entire available trajectory is used. Measuring the error in the fitting domain gives the *interpolation* error, which is artificially low in cases of overfitting, whereas the error in the verification window indicates the reliability of the extrapolated dipole moment. The error of the fit is estimated using one minus the coefficient of determination, *R*^2^, i.e.,
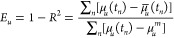
29where μ_*u*_^*m*^ is the mean value of the induced dipole moment. The error measure is unitless and independent of the magnitude of the dipole moment. The fitting method can be run in parallel with real-time simulations, which are terminated once *E*_*u*_ drops below a predefined threshold value. The computational cost of the fitting method is not insignificant, and we recommend that it is run once per time intervals of 50–100 a.u. when used to automatically terminate the real-time simulation.

Computing the error according to eq 29 provides an error estimate of the fit as a whole. In our experience from testing the algorithm with ideal multisinusoidal signals, the error of the fit depends primarily on the frequency estimation. Significant deviations in the estimated linear coefficients were only observed if there were frequencies missing or poorly estimated. In the case of ideal signals, there would be a significant error in the fit only if the frequency estimation failed. Real dipole data contain noise introduced by numerical integration in time. How this affects the distribution of error is unknown, although it is reasonable to believe that the main source of error still lies in the frequency estimation.

The error measure *E*_*u*_ cannot distinguish error contributions from different parts of the spectrum, preventing termination once the desired frequency region is converged. In order to focus on valence excitations in the low-frequency region, we apply a low-pass (smoothing) filter to remove frequencies above a cutoff frequency ω_max_ from the dipole moment in the time domain. We used a Butterworth filter, implemented by SciPy,^58^ which removes the high-energy part of the spectrum while leaving the lower-energy part almost unchanged. If bound core excitations are the main targets, then a high-pass filter must be used instead.

## 3 Computational Details

We test the dipole extrapolation scheme using RT-TDDFT simulations, supplemented by a few RT-TDCIS simulations, to demonstrate its applicability to wave function-based theories. The RT-TDDFT simulations are performed using the ReSpect program,^59^ while the RT-TDCIS calculations are performed using the Hylleraas Quantum Dynamics (HyQD) software.^60^ The RT-TDDFT and RT-TDCIS simulations are performed with analytic integration at *t* = 0 au, as described in ref (8). The subsequent time steps are performed numerically using the Magnus integrator for the RT-TDDFT simulations^8^ and the three-stage Gauss-Legendre integrator^61^ as described in ref (62) with the residual norm convergence criterion 10^–14^ a.u. for the implicit equations for the RT-TDCIS simulations.

The RT-TDCIS simulations are performed with time step *Δt* = 0.01 a.u. and field strength κ = 10^–3^ a.u. The RT-TDDFT simulations for the organic molecules CH_4_, CH_2_O, CH_3_OH, C_2_H_6_, and C_6_H_6_ are performed with time step *Δt* = 0.01 a.u. and field strength κ = 10^–4^ a.u., while *Δt* = 0.01 a.u. and κ = 10^–3^ a.u. are used for CO_2_, H_2_O, and NH_3_. For the smallest systems, He, H_2_, Be, and LiH, *Δt* = 0.1 a.u. and κ = 10^–3^ a.u. are used.

Molecular geometries are listed in the Supporting Information. The simulations were performed in Dunning’s cc-pVXZ and aug-cc-pVXZ, X = D,T, basis sets^63−65^ (uncontracted in the case of RT-TDDFT calculations). The RT-TDDFT simulations were performed using the PBE0 exchange–correlation potential^66−69^ in the adiabatic approximation.

Simulations are performed for all three Cartesian directions for all systems, even in cases in which point-group symmetry could have been easily exploited to reduce the computational effort to one or two directions. While this is mainly done to make automation simple (identical treatment for all systems), it also provides a simple check that the extrapolation algorithm does not significantly break point-group symmetry due to numerical noise in the input dipole trajectories.

Our implementation of the dipole-extrapolation algorithm is freely available at https://github.com/HyQD/absorption-spectrum.

## 4 Results

All reference spectra in this paper are produced from low-pass filtered electric dipole moments with a trajectory length of 4000 a.u., such that the spectral resolution becomes *Δω* = 1.6 · 10^–3^ a.u.. In this article, the resolution of the fitted spectrum *S̅*(ω) is the same as its reference spectrum. This is to allow direct comparisons of the two spectra, although the resolution of *S̅*(ω) could be made arbitrarily fine. The Fourier transform of the approximated dipole moment μ_*u*_ is calculated according to
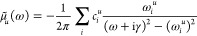
30The half-life parameter was always set to γ = 0.5 · 10^–3^π, and the spectra were cut at an estimated ionization energy of 0.5 a.u. – ϵ_HOMO_, where the energies of the highest occupied molecular orbital ϵ_HOMO_ of all systems are listed in the Supporting Information.

Using the low-pass filter will leave the lower energy part of the absorption spectrum unaltered, while the higher energy part is removed and set to zero. Differences between filtered and unfiltered spectra are shown in the Supporting Information. The low-pass filter does not give a clean cutoff at the cutoff frequency ω_max_ but rather a gradual lowering of the peak intensity around ω_max_. The cutoff frequency should therefore be set somewhat higher than the desired range of the spectrum. We have used ω_max_ = 4 a.u. for all systems.

When fitting the dipole moment, the available trajectory is from when the external field is turned off at *t* = 0 to time *t* = *T*_ver_^*u*^. The linear coefficients are determined on the time interval [0, *T*_fit_^*u*^], where *T*_fit_^*u*^ = 0.75*T*_ver_^*u*^. The frequencies are estimated on the entire available trajectory [0, *T*_ver_^*u*^] but with a limit on the total number of data points supplied to the Padé, set to 5 · 10^3^. The reduction in points, if exceeding the limit, is done by effectively increasing the time step *Δt* used (by an integer factor) when creating the Padé. The error *E*_*u*_ is evaluated only on (*T*_fit_^*u*^, *T*_ver_^*u*^]. The error in spectrum *E*_*S*_ is calculated the same way as that in the time domain, as given in eq 29.

### 4.1 Performance on a Selection of Systems

For each spatial direction, the convergence of the fit is tested every 50 au in time of the trajectory length, starting from *T*_min_ = 100 a.u.. The simulation is terminated when the fit has converged below a given threshold or when the trajectory length reaches *T*_max_ = 1000 a.u., which corresponds to a target minimum spectral resolution of 0.006 a.u.. The convergence criterion was set to *E*_*u*_ < 10^–3^, a strict threshold corresponding to a near-perfect fit. The criterion was set based on preliminary investigations.^48^ Since the real-time calculations on a given system using three spatial directions of the external field are independent, the trajectory length needed for a converged fit might vary between the three simulations.

The required trajectory length of each spatial direction *T*_ver_^*u*^ and their corresponding verification error *E*_*u*_ as well as the error in the spectrum *E*_*S*_ are listed in Tables 1 and 2. The fitting of the dipole moment from RT-TDCIS calculations is shown in Table 1, while the fitting of the RT-TDDFT data is found in Table 2. Figures of the approximated spectra of all systems can be found in the Supporting Information.

**Table 1:** Convergence Times and Corresponding Errors of Systems from RT-TDCIS Calculations

	basis	*T*_ver_^*x*^ [a.u.]	*T*_ver_^*y*^ [a.u.]	*T*_ver_^*z*^ [a.u.]	*E*_*x*_	*E*_*y*_	*E*_*z*_	*E*_*S*_
CH_2_O	aug-cc-pVDZ	450	600	650	8 · 10^–6^	8 · 10^–4^	1 · 10^–3^	1 · 10^–3^
CO_2_	cc-pVDZ	100	100	100	8 · 10^–5^	8 · 10^–5^	3 · 10^–6^	2 · 10^–4^
	aug-cc-pVDZ	250	250	200	6 · 10^–5^	6 · 10^–5^	3 · 10^–4^	2 · 10^–3^
	aug-cc-pVTZ	300	300	250	2 · 10^–4^	2 · 10^–4^	1 · 10^–4^	2 · 10^–3^
H_2_O	aug-cc-pVDZ	150	200	300	1 · 10^–5^	2 · 10^–4^	7 · 10^–5^	3 · 10^–4^
NH_3_	aug-cc-pVDZ	350	300	300	4 · 10^–5^	7 · 10^–4^	7 · 10^–6^	3 · 10^–3^

**Table 2:** Convergence Times and Corresponding Errors of Systems from RT-TDDFT Calculations

	basis	*T*_ver_^*x*^ [a.u.]	*T*_ver_^*y*^ [a.u.]	*T*_ver_^*z*^ [a.u.]	*E*_*x*_	*E*_*y*_	*E*_*z*_	*E*_*S*_
Be	aug-ucc-pVTZ	100	100	100	1 · 10^–8^	1 · 10^–8^	1 · 10^–8^	6 · 10^–6^
C_2_H_6_	aug-ucc-pVDZ	750	800	550	6 · 10^–4^	7 · 10^–4^	5 · 10^–4^	8 · 10^–4^
	aug-ucc-pVTZ	1000	1000	950	5 · 10^–2^	3 · 10^–2^	2 · 10^–5^	6 · 10^–3^
C_6_H_6_	aug-ucc-pVDZ	1000	1000	550	3 · 10^–2^	4 · 10^–2^	7 · 10^–4^	4 · 10^–2^
CH_2_O	aug-ucc-pVDZ	450	650	700	4 · 10^–6^	8 · 10^–4^	1 · 10^–4^	5 · 10^–4^
	aug-ucc-pVTZ	650	900	1000	2 · 10^–4^	4 · 10^–4^	2 · 10^–3^	8 · 10^–4^
CH_3_OH	aug-ucc-pVDZ	1000	1000	1000	2 · 10^–1^	8 · 10^–1^	4 · 10^–2^	8 · 10^–2^
	aug-ucc-pVTZ	1000	1000	1000	3 · 10^–1^	8 · 10^–1^	4 · 10^–1^	2 · 10^–1^
CH_4_	aug-ucc-pVDZ	200	200	200	8 · 10^–4^	7 · 10^–4^	6 · 10^–4^	2 · 10^–3^
	aug-ucc-pVTZ	350	350	350	4 · 10^–4^	3 · 10^–4^	3 · 10^–4^	3 · 10^–3^
CO_2_	aug-ucc-pVDZ	350	350	250	4 · 10^–4^	4 · 10^–4^	1 · 10^–4^	5 · 10^–4^
H_2_O	aug-ucc-pVDZ	200	250	300	2 · 10^–6^	3 · 10^–6^	3 · 10^–4^	3 · 10^–4^
H_2_	aug-ucc-pVTZ	100	100	100	1 · 10^–7^	1 · 10^–7^	8 · 10^–8^	5 · 10^–6^
He	aug-ucc-pVTZ	100	100	100	9 · 10^–8^	9 · 10^–8^	1 · 10^–7^	9 · 10^–6^
LiH	aug-ucc-pVDZ	100	100	300	1 · 10^–5^	3 · 10^–5^	3 · 10^–4^	3 · 10^–4^
NH_3_	aug-ucc-pVDZ	350	400	300	2 · 10^–4^	4 · 10^–4^	7 · 10^–4^	1 · 10^–3^

The fitting method reached the strict threshold for most systems with a maximum spectral error of *E*_*S*_ ≤ 3 · 10^–3^. For all converged systems, the approximated functions for dipole moment μ_*u*_(*t*) reliably reproduce its reference spectrum. The systems with very sparse spectra (He, H_2_, and Be) converged instantly (*T*_ver_ = 100 a.u.), providing approximated spectra indistinguishable from their reference spectra. In these cases, the fitting method achieved a speedup of 10 times compared to the max trajectory length of *T*_max_ = 1000 a.u. or 40 times compared to computing the reference spectra (using 4000 a.u.). The reduction in computational cost achieved by using the fitting method is relative to the desired spectral resolution. We would argue that the least unambiguous way to assess the speedup is to compare the convergence times *T*_ver_^*u*^ with the simulation time that would have been used if the fitting method was not used, i.e., the max trajectory length. Should the method converge at the preset max trajectory length, one may argue that no simulation time was spared. In this case, one still achieves arbitrary improvement in the spectral resolution.

Systems with relatively sparse spectra (CH_4_, CO_2_, H_2_O, LiH, and NH_3_) also converged nicely with short dipole trajectories (*T*_ver_^*u*^ ≤ 350 a.u.). As the spectral density increases, the fitting method struggles to converge. Systems like C_2_H_6_ and CH_2_O only converged when using a double-zeta basis set, while the fitting of C_6_H_6_ and CH_3_OH did not achieve errors below the low threshold.

**Figure 1:**
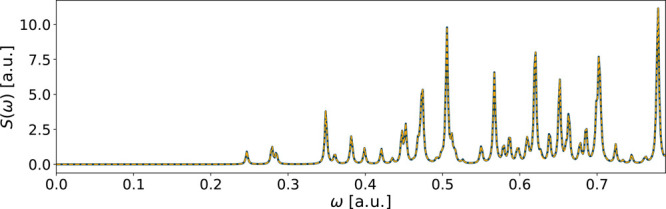
Spectrum of CH_2_O using the aug-ucc-pVDZ basis in an RT-TDDFT simulation. The reference spectrum is in solid blue, while the yellow dashed line shows the spectrum of the fitted functions. The fitting errors are *E*_*x*_ = 4 · 10^–6^, *E*_*y*_ = 8 · 10^–4^, and *E*_*z*_ = 1 · 10^–4^.

The CH_2_O molecule with a double-zeta basis set converged for both of the real-time methods. The spectra of the fit in both cases are nearly indistinguishable from their reference spectra. A comparison between the approximated and reference spectra from RT-TDDFT calculations is shown in Figure 1. The RT-TDDFT triple-zeta case nearly reached the error threshold (*E*_*z*_ = 2 · 10^–3^), also providing a very low spectral error (*E*_*S*_ = 8 · 10^–4^).

**Figure 2:**
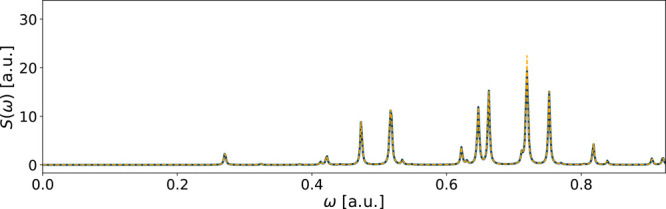
Spectrum of NH_3_ using the aug-cc-pVDZ basis in an RT-TDCIS simulation. The reference spectrum is in solid blue, while the yellow dashed line shows the spectrum of the converged fitted functions. This was the poorest approximated spectrum of all converged cases. The fitting errors are *E*_*x*_ = 4 · 10^–5^, *E*_*y*_ = 7 · 10^–4^, and *E*_*z*_ = 7 · 10^–6^.

Among the converged systems, NH_3_ from RT-TDCIS calculations showed the largest error compared to its reference spectrum (*E*_*S*_ = 3 · 10^–3^). Its spectrum is shown in Figure 2, and it was the approximated spectrum with the most visible deviation from its reference spectrum among the converged systems. The approximated spectrum shows a deviation in a peak at ω ≈ 0.75 a.u., but the rest of the peaks correspond well to the reference spectrum.

**Figure 3:**
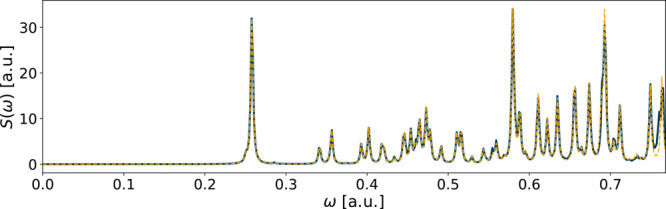
Spectrum of C_6_H_6_ using the aug-ucc-pVDZ basis in an RT-TDDFT simulation. The reference spectrum is in solid blue, while the yellow dashed line shows the spectrum of the fitted functions. The fitting errors are *E*_*x*_ = 3 · 10^–2^, *E*_*y*_ = 4 · 10^–2^, and *E*_*z*_ = 7 · 10^–4^.

The fitting method only partially converged for C_6_H_6_, as well as C_2_H_6_ and CH_2_O with triple-zeta basis, meaning that the error of the fit was below the set threshold in only one or two of the spatial directions. Still, the spectral error in all three cases is quite low. The result of the fitting of benzene is shown in Figure 3, which had the largest spectral error (*E*_*S*_ = 4 · 10^–2^) of the three.

The trajectory length needed for the fitting method to converge strongly depends on the spectral density. We observed a trend in that fitting becomes increasingly difficult as the spectral density increases. Increasing either the number of electrons in the system or the size of the basis set will, in general, require longer real-time simulations before the fitting method converges. The trend with increasing basis set size is clearly seen from the fitting of CO_2_ from RT-TDCIS calculations. The simulation using the cc-pVDZ basis set converges faster (*T*_ver_^*u*^ = 100 a.u.) than when using the larger basis sets like the aug-cc-pVDZ basis set (*T*_ver_^*u*^ ≤ 250 a.u.) or aug-cc-pVTZ basis set (*T*_ver_^*u*^ ≤ 300 a.u.).

**Figure 4:**
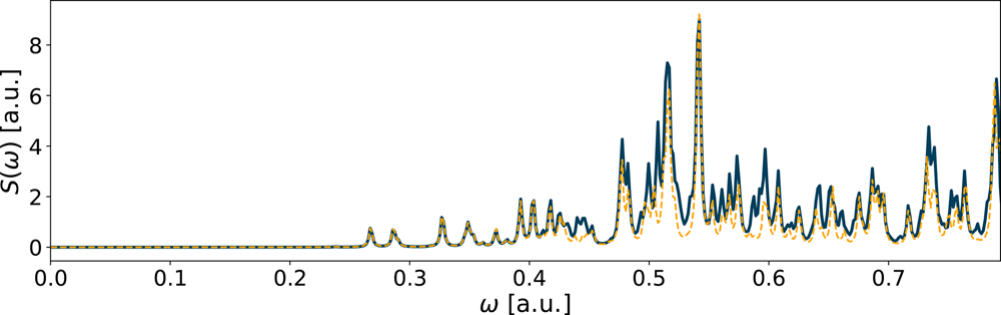
Spectrum of CH_3_OH using the aug-ucc-pVTZ basis in an RT-TDDFT simulation. The reference spectrum is in solid blue, while the yellow dashed line shows the spectrum of the fitted functions. The fitting errors are *E*_*x*_ = 3 · 10^–1^, *E*_*y*_ = 8 · 10^–1^, and *E*_*z*_ = 4 · 10^–1^.

Only the fit of CH_3_OH did not result in errors below the convergence threshold in any of the spatial directions. This was true for both the double and triple-zeta basis sets (from RT-TDDFT calculations). The result using a triple-zeta basis set is shown in Figure 4 and is the case with the highest error in the time domain, *E*_*u*_ ∼ 10^–1^. There is a significant deviation from the reference spectrum, although the main features are intact. Of all systems in this paper, this gave the worst approximation to the reference spectrum. Despite this, the spectrum *S̅*(ω) still provides a reasonable coarse approximation.

These results are promising in all cases, as the converged fit seems to reproduce its reference spectrum reliably with only minor deviations in the peak intensities. The error of the fit *E*_*u*_ also correlates with the spectral error, *E*_*S*_. This predictability is crucial if the convergence criterion is used to automatically terminate real-time simulations. Our results also indicate that the convergence criterion used in this study is stricter than necessary. A slight relaxation in the criterion might lead to faster convergence without significantly impacting the quality of the approximated spectrum.

For the estimated dipole moment, the frequencies and their corresponding linear coefficients are known. For a successful fit, one may therefore obtain the transition probability |⟨0|μ̂_*u*_|*n*⟩|^2^ of a transition with energy *E*_*n*_ – *E*_0_ directly from the linear coefficient, as |⟨0|μ̂_*u*_|*n*⟩|^2^ = *B*_*n*_^*u*^/(2κ). This could be used to calculate the oscillator strength and create stick spectra. However, estimated frequencies in different spatial directions but corresponding to the same transition will have a small error associated with the frequencies. In order to compute the oscillator strength, one would therefore have to assess which estimated frequencies across the spatial directions correspond to the same transition.

The convergence of the dipole moment fitting depends primarily on the frequency estimation. When the fit does not converge, it follows that the Fourier–Padé approximant is not sufficiently converged to accurately capture the Bohr frequencies. The quality of the Fourier–Padé depends on the dipole trajectory length *t*_*N*_ rather than the number of steps or step length.^51^ However, there is no given final time *t*_*N*_ ensuring convergence; the necessary trajectory length depends on the spectral density. High spectral density can cause the Fourier–Padé to fail, even for relatively long simulations. The general Padé approximant is prone to instabilities due to problems with near-degeneracy of the linear system. As pointed out by Cooper et al.,^70^ the Fourier–Padé used in real-time spectroscopy is known to struggle with dense spectra. The fitting method introduces a measure of the error *E*_*u*_ that does not rely on any reference spectrum. This introduces a more reliable way of estimating the error in the approximated spectrum.

### 4.2 Fitting Using MO Decomposition

We assessed the performance of the fitting procedure used to extrapolate the components of the dipole moment of C_6_H_6_ decomposed to the MO pairs μ_*u*_^*ia*^ in the RT-TDDFT calculation. Instead of creating a fitting function for each individual MO pair, which would increase the memory overhead, we clustered the components μ_*u*_^*ia*^ into groups of ten. These groups are formed so that the overall sparsity of the spectra obtained for each cluster is maintained. This is accomplished by spreading the individual constituents of the cluster across the energy range. A fitted function of each cluster was then created from the sum of the MO pairs that the cluster contains. The total error is measured for the full dipole moment, μ_*u*_(*t*). Some of the MO pairs were omitted entirely, making the MO decomposition work as a low-pass filter. When fitting components, the low-pass filter is, therefore, not needed.

Fitting the decomposed signal, however, did not improve the convergence compared with when the full dipole moment was used for extrapolation. Simulations for both directions μ_*x*_ and μ_*y*_ reached the max trajectory length (*t* = 1000 a.u.) without the fitting error going below the error threshold. The errors (*E*_*x*_ = 1 · 10^–2^ and *E*_*y*_ = 1 · 10^–2^) were only slightly lower compared with fitting without the MO decomposition. The last spatial direction μ_*z*_ converged at *T*_ver_ = 650 a.u. (*E*_*z*_ = 4 · 10^–4^), which is somewhat slower than without MO decomposition. The spectral error was *E*_*S*_ = 9 · 10^–3^, which corresponds to a low spectral error.

**Figure 5:**
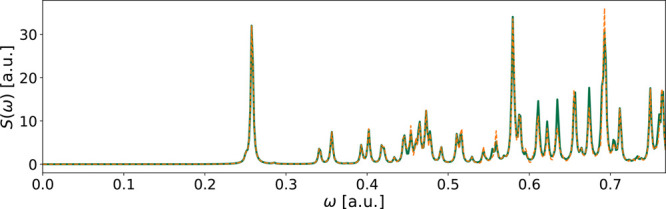
Spectrum of C_6_H_6_ using the aug-ucc-pVDZ basis in a RT-TDDFT simulation. The reference spectrum is in solid green, while the orange dashed line shows the spectrum of the fitted functions using molecular orbital decomposition from *T*_ver_^*u*^ = 600 a.u..

**Figure 6:**
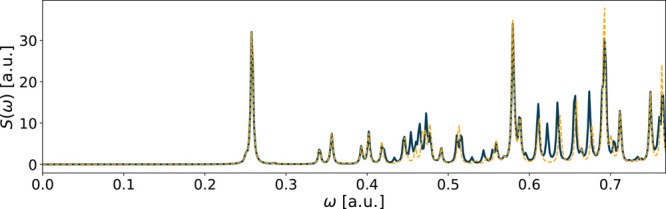
Spectrum of C_6_H_6_ using the aug-ucc-pVDZ basis in a RT-TDDFT simulation. The reference spectrum is in solid blue, while the yellow dashed line shows the spectrum of the fitted functions from *T*_ver_^*u*^ = 600 a.u..

Although the MO decomposition did not lead to an accelerated convergence of the fitting method, we still observed improvements. For example the simulation with *T*_ver_^*u*^ = 600 a.u. has a lower error of the fit for the decomposed dipole moment (*E*_*x*_ = 6 · 10^–2^, *E*_*y*_ = 4 · 10^–2^, and *E*_*z*_ = 1 · 10^–3^) for all spatial directions compared to the fit on the full dipole moment, (*E*_*x*_ = *E*_*y*_ = 3 · 10^–1^ and *E*_*z*_ = 2 · 10^–3^). The decomposed fit in Figure 5 (*E*_*S*_ = 3 · 10^–2^) is visibly improved compared to the fit using the full dipole moment in Figure 6 (*E*_*S*_ = 2 · 10^–1^).

It is important to note that the scope of our testing of the fitting method using MO decomposition was limited. Previous success using the Fourier–Padé approximant in combination with the MO decomposition on RT-TDDFT data suggests that this in many cases is very effective.^22^ Our study, however, raises cause for caution regarding the use of the Fourier–Padé approximant: The Padé *can* struggle, even when using MO decomposition. The unknown amount of error introduced to the final spectrum by this procedure remains an open problem that the user should be aware of when analyzing spectra with the Fourier–Padé method with MO decomposition.

The particular form of components μ_*u*_^*pq*^ is also very dependent on the quantum mechanical method used to compute the time-dependent wave function. Using MO decomposition on the electric dipole moment from RT-TDCCSD calculations leads to large overlaps in frequencies among different components.^48^ The usefulness of such decomposition might vary among the different quantum mechanical frameworks.

## 5 Conclusion

We have developed a novel method for creating functions approximating the electric dipole moment from real-time calculations. The fitted functions for the dipole moment in the three spatial directions can then be used to produce absorption spectra with an arbitrary high resolution. Real-time calculations of absorption spectra require the use of discrete Fourier transforms, demanding long simulation times to obtain high spectral resolution. In our work, we have shown that the length of the real-time simulations and, hence, the computational cost can be greatly reduced by the developed fitting method.

We introduced a quantitative error measure to evaluate the convergence of the fit. For all systems in this work, a converged fit reliably reproduced the reference spectrum from long real-time calculations. A convergence criterion of 10^–3^ seems to be quite strict, and further studies should be conducted to investigate the impact of slightly higher errors on the estimated spectrum. In order to reduce the computational cost of calculating absorption spectra, real-time calculations should be automatically terminated once the convergence criterion is reached.

In this work, we set the verification window to 25% of the available dipole trajectory. The critical step of the method is determining the frequencies, which always uses all available data. For the linear optimization, the verification window should include an entire period of the smallest frequency in the signal as an insufficiently large verification window may lead to misleading error estimates. In future work, the verification window should depend on an estimate of the smallest frequency in the signal based on differences in the molecular orbital energies.

The fitting method converged with as little as 100 a.u. long trajectories in time for systems with sparse spectra. Convergence slows as spectral density increases, even leading to failure of convergence in some cases. The current version of the fitting method shows encouraging results for smaller systems, although aspects of the method require further investigation.

Our testing of the fitting method using the molecular orbital decomposition of a single system gave mixed results. The decomposition did not enable the fit to meet the convergence criterion, although we observed improvements in the approximated spectrum. This motivates the need for further investigations.

An apparent weakness of the current implementation is the way of estimating frequencies. Future versions should not rely on Fourier–Padé but rather investigate other methods of estimating frequencies. This could include other methods for harmonic inversion or letting the function form of the fitted dipole moment be a truncated Fourier series based on an estimation of the fundamental frequency. The same frequencies can appear in all spatial directions, which could be exploited to improve frequency estimation. In particular, in cases where the frequencies are successfully estimated in one spatial direction, knowledge of these existing frequencies could be used to alleviate the search in other spatial directions with potentially higher spectral density. Improving the frequency estimation is crucial for stabilizing the fitting method for systems with a high spectral density.

This work has focused on the Dirac delta impulse, although the general fitting algorithm may be used on systems with any type of external field. Using a laser pulse targeting a specific spectral region may provide both an upper and lower bound when estimating the frequencies. Any *a priori* information about the frequencies should be exploited by the fitting algorithm. Additionally, the Dirac delta impulse targets *all* excitation energies, thereby maximizing the spectral density. It is not unlikely that a narrow-band laser pulse would somewhat alleviate the fitting process by reducing the spectral density.

## Supporting Information Available

Molecular geometries and HOMO energies for all systems. Comparisons between the spectra of filtered versus unfiltered dipole moments. Spectra of the fitted functions of the dipole moments compared with the corresponding reference spectrum (PDF)
