# Supplementary material for: Cost-Efficient High-Resolution Linear Absorption Spectra through Extrapolating the Dipole Moment from Real-Time Time-Dependent Electronic-Structure Theory
Source: J Chem Theory Comput. 2023 Oct 24;19(21):7764–75. doi: 10.1021/acs.jctc.3c00727 (PMC10653104; doi:10.1021/acs.jctc.3c00727)
Supplement: Supplementary file 1 — ct3c00727_si_001.pdf [file ct3c00727_si_001.pdf]

Supporting Information for "Cost-Efficient High-Resolution Linear Absorption Spectra Through Extrapolating the Dipole Moment from Real-Time Time-Dependent Electronic-Structure Theory"

Eirill Hauge,[†,‡] Håkon Emil Kristiansen,[†] Lukas Konecny,[¶,§] Marius Kadek,[¶,‖] Michal Repisky,[¶,⊥] and Thomas Bondo Pedersen[*,†]

†*Hylleraas Centre for Quantum Molecular Sciences, Department of Chemistry, University of Oslo, P.O. Box 1033, Blindern, 0315 Oslo, Norway*

‡*Department of Numerical Analysis and Scientific Computing, Simula Research Laboratory, Kristian Augusts gate 23, 0164 Oslo, Norway*

¶*Hylleraas Centre for Quantum Molecular Sciences, Department of Chemistry, University of Tromsø—The Arctic University of Norway, N-9037 Tromsø, Norway*

§*Max Planck Institute for the Structure and Dynamics of Matter, Center for Free Electron Laser Science, Luruper Chaussee 149, 22761 Hamburg, Germany*

‖*Department of Physics, Northeastern University, Boston, Massachusetts 02115, USA*

⊥*Department of Physical and Theoretical Chemistry, Faculty of Natural Sciences, Comenius University, Ilkovicova 6, SK-84215 Bratislava, Slovakia*

E-mail: t.b.pedersen@kjemi.uio.no

S1 Molecular geometries

The molecular geometries of all systems used in the numerical study are listed in Tables S1 to S10. The same geometries were used in both the real-time time-dependent density-functional theory (RT-TDDFT) and the real-time time-dependent configuration interaction singles (RT-TDCIS) calculations.

Table S1: Molecular geometry of C_2H_6.

	x [Å]	y [Å]	z [Å]
C	0.0000	0.0000	0.7610
	0.0000	0.0000	−0.7610
H	0.0000	1.0233	1.1616
	−0.8862	−0.5116	1.1616
	0.8862	−0.5116	1.1616
	0.0000	−1.0233	−1.1616
	−0.8862	0.5116	−1.1616
	0.8862	0.5116	−1.1616

Table S2: Molecular geometry of C_6H_6.

	x [Å]	y [Å]	z [Å]
C	0.0000	1.3949	0.0000
	1.2080	0.6975	0.0000
	1.2080	−0.6975	0.0000
	0.0000	−1.3949	0.0000
	−1.2080	−0.6975	0.0000
	−1.2080	0.6975	0.0000
H	0.0000	2.4858	0.0000
	2.1528	1.2429	0.0000
	2.1528	−1.2429	0.0000
	0.0000	−2.4858	0.0000
	−2.1528	−1.2429	0.0000
	−2.1528	1.2429	0.0000

Each system's highest occupied molecular orbital energy is listed in Tables S11 and S12, for the calculations using RT-TDDFT and RT-TDCIS respectively.

Table S3: Molecular geometry of CH_2O.

	x [Å]	y [Å]	z [Å]
O	0.0000	0.0000	0.6751
C	0.0000	0.0000	−0.5280
H	0.0000	0.9462	−1.1162
	0.0000	−0.9462	−1.1162

Table S4: Molecular geometry of CH_3OH.

	x [Å]	y [Å]	z [Å]
C	−0.0463	0.6610	0.0000
O	−0.0463	−0.7546	0.0000
H	−1.0964	0.9762	0.0000
	0.4402	1.0772	0.8975
	0.4402	1.0772	−0.8975
	0.8645	−1.0602	0.0000

Table S5: Molecular geometry of CH_4.

	x [Å]	y [Å]	z [Å]
C	0.0000	0.0000	0.0000
H	0.6328	0.6328	0.6328
	−0.6328	−0.6328	0.6328
	−0.6328	0.6328	−0.6328
	0.6328	−0.6328	−0.6328

Table S6: Molecular geometry of CO_2.

	x [Å]	y [Å]	z [Å]
C	0.0000	0.0000	0.0000
O	0.0000	0.0000	1.1363
	0.0000	0.0000	−1.1363

Table S7: Molecular geometry of H_2O.

	x [Å]	y [Å]	z [Å]
O	0.0000	0.0000	−0.0656
H	0.0000	0.7567	0.5203
	0.0000	−0.7567	0.5203

Table S8: Molecular geometry of LiH.

	x [Å]	y [Å]	z [Å]
Li	0.0000	0.0000	0.0000
H	0.0000	0.0000	1.6299

Table S9: Molecular geometry of H_2.

	x [Å]	y [Å]	z [Å]
H	0.0000	0.0000	0.0000
	0.0000	0.0000	0.7408

Table S10: Molecular geometry of NH_3.

	x [Å]	y [Å]	z [Å]
N	0.0000	0.0000	0.1064
H	0.0000	0.9335	−0.2482
	0.8084	−0.4667	−0.2482
	−0.8084	−0.4667	−0.2482

Table S11: Highest occupied molecular orbital energy of the systems using DFT calculations.

	basis	ϵ_{HOMO} [a.u.]
Be	aug-ucc-pVTZ	−0.2386
C_2H_6	aug-ucc-pVDZ	−0.3506
	aug-ucc-pVTZ	−0.3509
C_6H_6	aug-ucc-pVDZ	−0.2681
CH_2O	aug-ucc-pVDZ	−0.2887
	aug-ucc-pVTZ	−0.2894
CH_3OH	aug-ucc-pVDZ	−0.2925
	aug-ucc-pVTZ	−0.2935
CH_4	aug-ucc-pVDZ	−0.4024
	aug-ucc-pVTZ	−0.4026
CO_2	aug-ucc-pVDZ	−0.3947
H_2O	aug-ucc-pVDZ	−0.3333
H_2	aug-ucc-pVTZ	−0.4412
He	aug-ucc-pVTZ	−0.6695
LiH	aug-ucc-pVDZ	−0.1992
NH_3	aug-ucc-pVDZ	−0.2807

Table S12: Highest occupied molecular orbital energy of the systems using CIS calculations.

	basis	ϵ_{HOMO} [a.u.]
CH_2O	aug-cc-pVDZ	−0.4345
CO_2	cc-pVDZ	−0.5372
	aug-cc-pVDZ	−0.5459
	aug-cc-pVTZ	−0.5463
H_2O	aug-cc-pVDZ	−0.5095
NH_3	aug-cc-pVDZ	−0.4253

S2 Filtering the dipole moment

The reference spectra are filtered using a low-pass filter. The comparisons between the filtered and unfiltered spectra are shown in Figs. S1 to S4 and S6 to S23, contrasting the filtered spectra (dark blue line) with the parts which have been filtered (faded blue line). The cut-off frequency was set to $\omega_{\text{max}} = 4\,\text{a.u.}$, which is safely higher than what could be expected values for ionization energies. As can be seen from the figures, the low-pass filter does not provide a clean cut in the spectrum. Peaks around ω_{max} have lower intensity, also on the left side in the spectra.

The figures showing the filtering of spectra from the RT-TDDFT calculations are shown in Figs. S1 to S4 and S6 to S17, while Figs. S18 to S23 shows the filtering of spectra from the RT-TDCIS calculations.

The effects of the discarding high-energy molecular orbital transitions on the spectrum of C_6H_6 using the aug-ucc-pVDZ basis set in a RT-TDDFT calculation is shown in Fig. S5. Here, the spectrum of the molecular orbital components (dark green line) is compared to the full unfiltered spectrum (faded blue line).

S3 Spectra of the approximated functions

The figures in Figs. S41 to S46 compares the spectrum of the fitted function (yellow dashed line) with its corresponding reference spectrum (solid dark blue line). The convergence

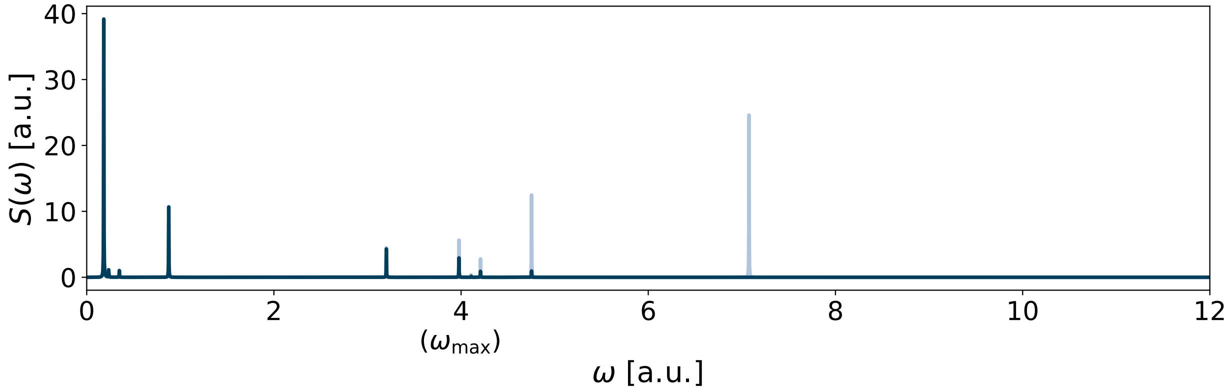

Figure S1: Effects of filtering the absorption spectrum of Be using the aug-ucc-pVTZ basis in a RT-TDDFT simulation. The dark line shows the filtered spectrum, while the faded line shows the unfiltered spectrum.

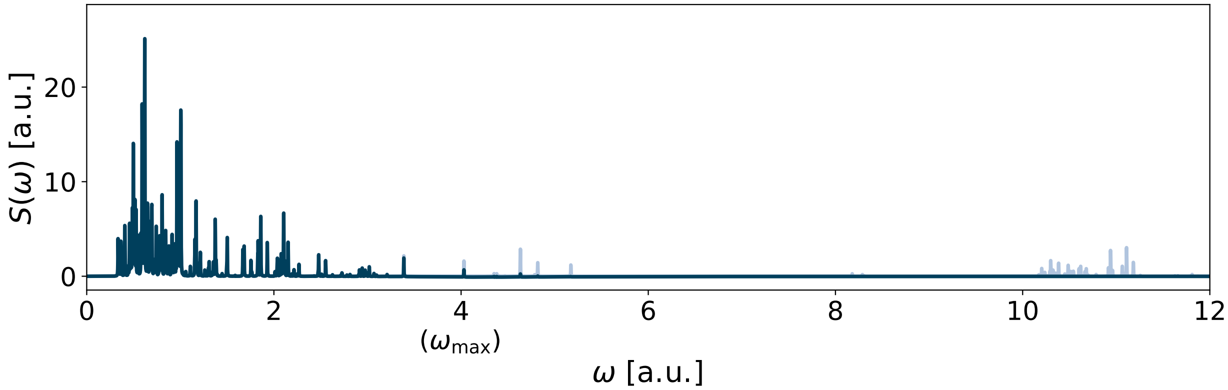

Figure S2: Effects of filtering the absorption spectrum of C_2H_6 using the aug-ucc-pVDZ basis in a RT-TDDFT simulation. The dark line shows the filtered spectrum, while the faded line shows the unfiltered spectrum.

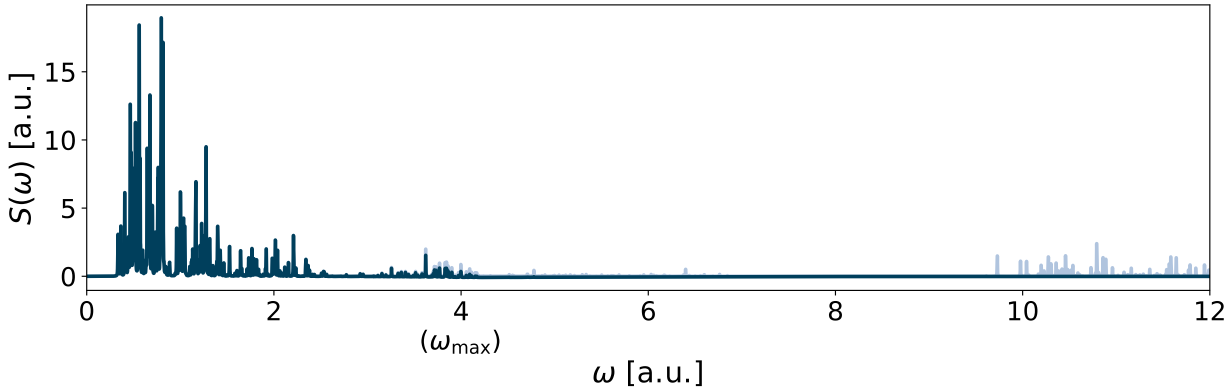

Figure S3: Effects of filtering the absorption spectrum of C_2H_6 using the aug-ucc-pVTZ basis in a RT-TDDFT simulation. The dark line shows the filtered spectrum, while the faded line shows the unfiltered spectrum.

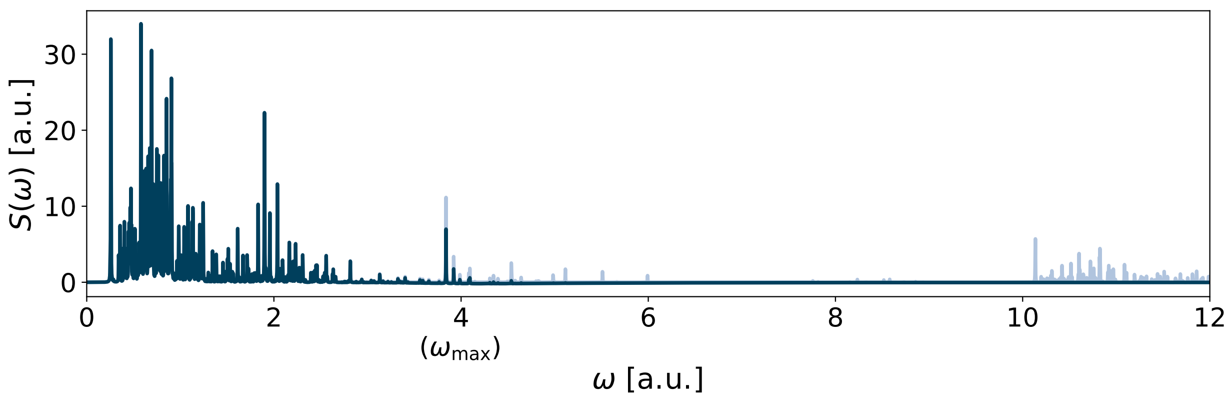

Figure S4: Effects of filtering the absorption spectrum of C_6H_6 using the aug-ucc-pVDZ basis in a RT-TDDFT simulation. The dark line shows the filtered spectrum, while the faded line shows the unfiltered spectrum.

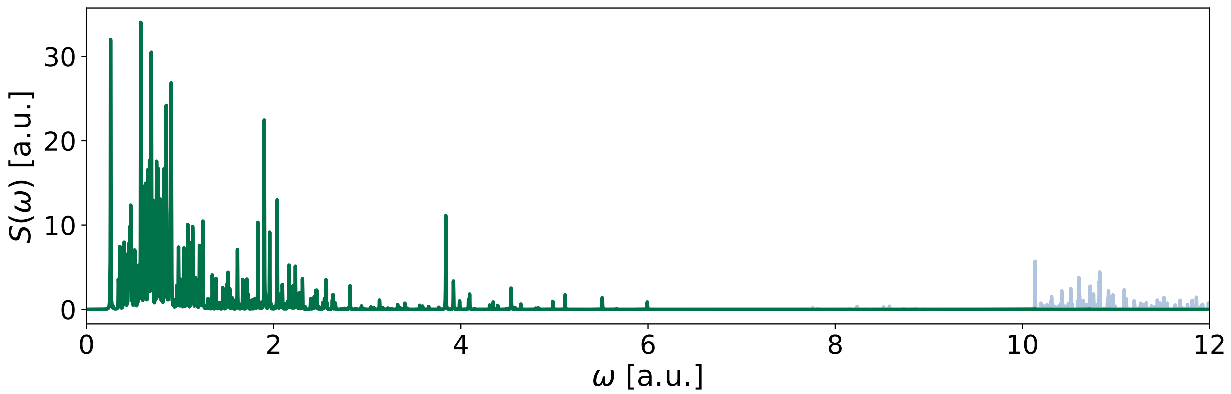

Figure S5: Effects of discarding high-energy molecular orbital transitions on the absorption spectrum of C_6H_6 using the aug-ucc-pVDZ basis in a RT-TDDFT simulation. The dark green line shows the spectrum of the sum of the molecular orbital pair components, while the faded line shows the full unfiltered spectrum.

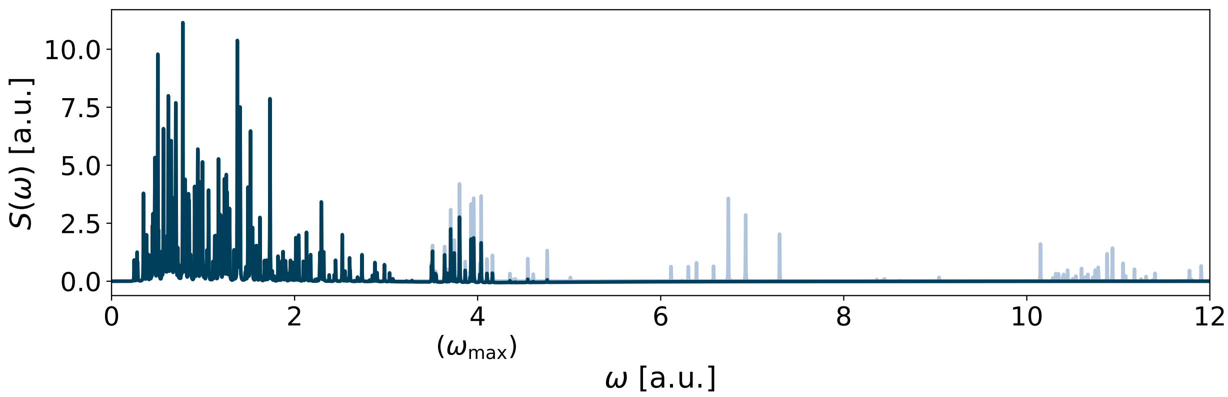

Figure S6: Effects of filtering the absorption spectrum of CH_2O using the aug-ucc-pVDZ basis in a RT-TDDFT simulation. The dark line shows the filtered spectrum, while the faded line shows the unfiltered spectrum.

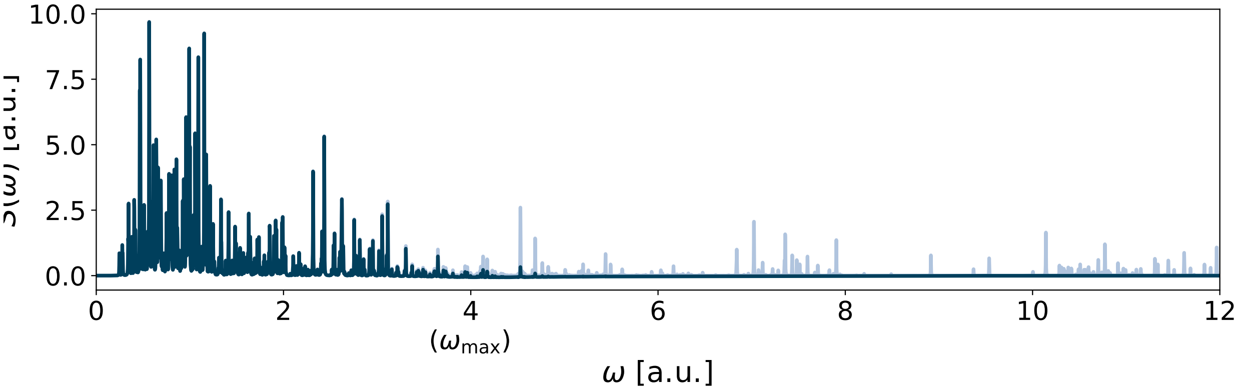

Figure S7: Effects of filtering the absorption spectrum of CH_2O using the aug-ucc-pVTZ basis in a RT-TDDFT simulation. The dark line shows the filtered spectrum, while the faded line shows the unfiltered spectrum.

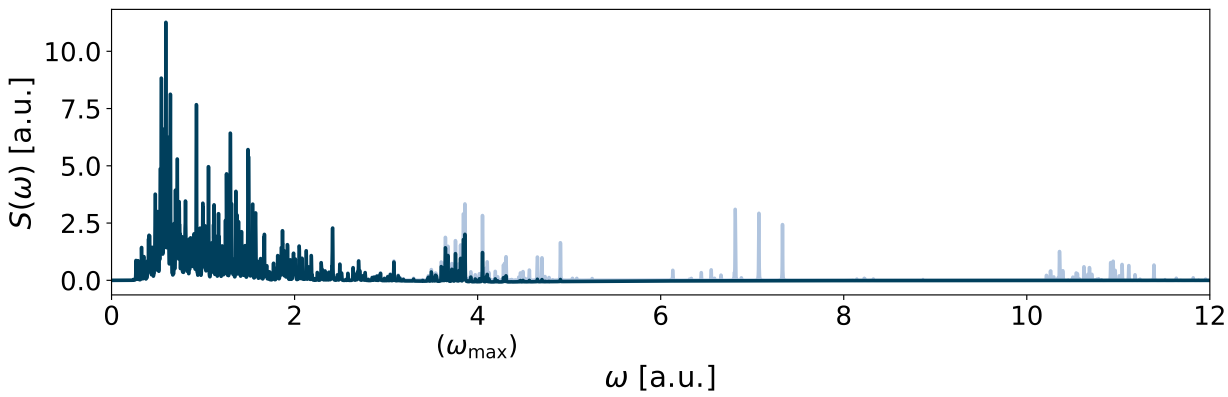

Figure S8: Effects of filtering the absorption spectrum of CH_3OH using the aug-ucc-pVDZ basis in a RT-TDDFT simulation. The dark line shows the filtered spectrum, while the faded line shows the unfiltered spectrum.

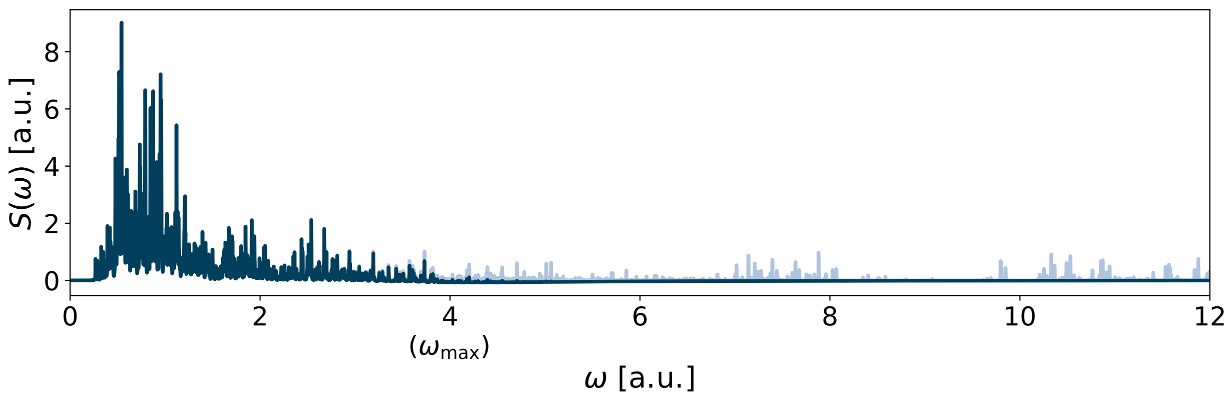

Figure S9: Effects of filtering the absorption spectrum of CH_3OH using the aug-ucc-pVTZ basis in a RT-TDDFT simulation. The dark line shows the filtered spectrum, while the faded line shows the unfiltered spectrum.

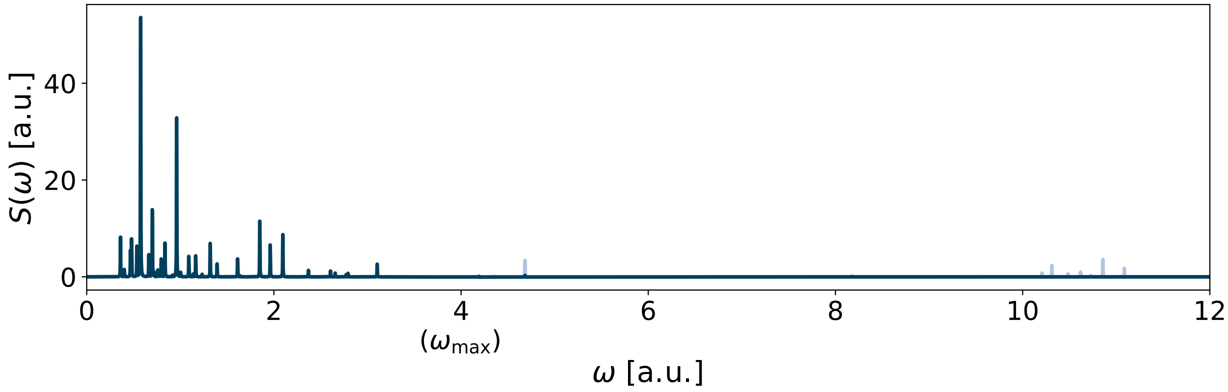

Figure S10: Effects of filtering the absorption spectrum of CH_4 using the aug-ucc-pVDZ basis in a RT-TDDFT simulation. The dark line shows the filtered spectrum, while the faded line shows the unfiltered spectrum.

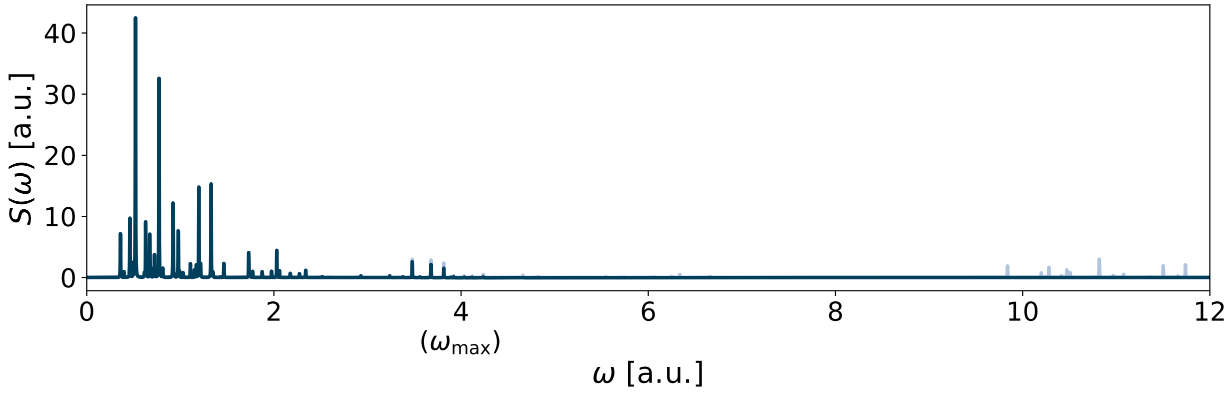

Figure S11: Effects of filtering the absorption spectrum of CH_4 using the aug-ucc-pVTZ basis in a RT-TDDFT simulation. The dark line shows the filtered spectrum, while the faded line shows the unfiltered spectrum.

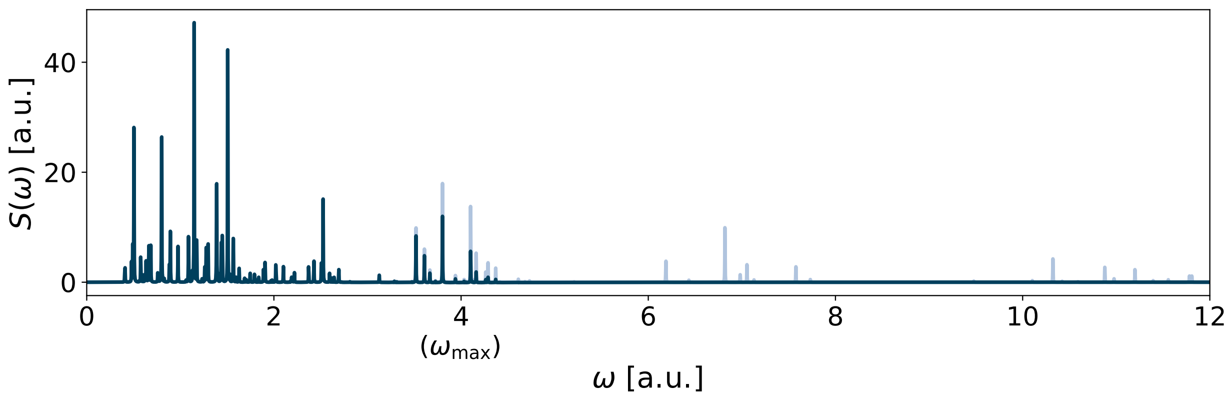

Figure S12: Effects of filtering the absorption spectrum of CO_2 using the aug-ucc-pVDZ basis in a RT-TDDFT simulation. The dark line shows the filtered spectrum, while the faded line shows the unfiltered spectrum.

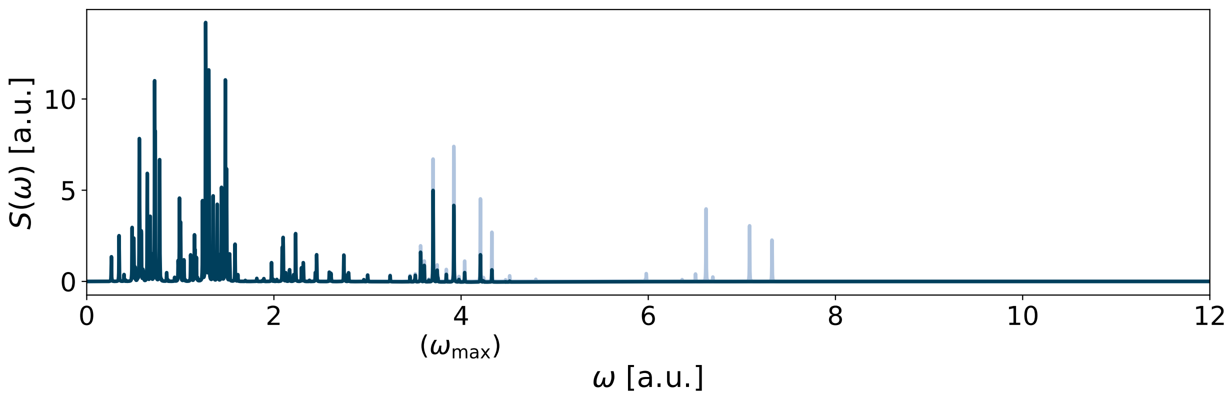

Figure S13: Effects of filtering the absorption spectrum of H_2O using the aug-ucc-pVDZ basis in a RT-TDDFT simulation. The dark line shows the filtered spectrum, while the faded line shows the unfiltered spectrum.

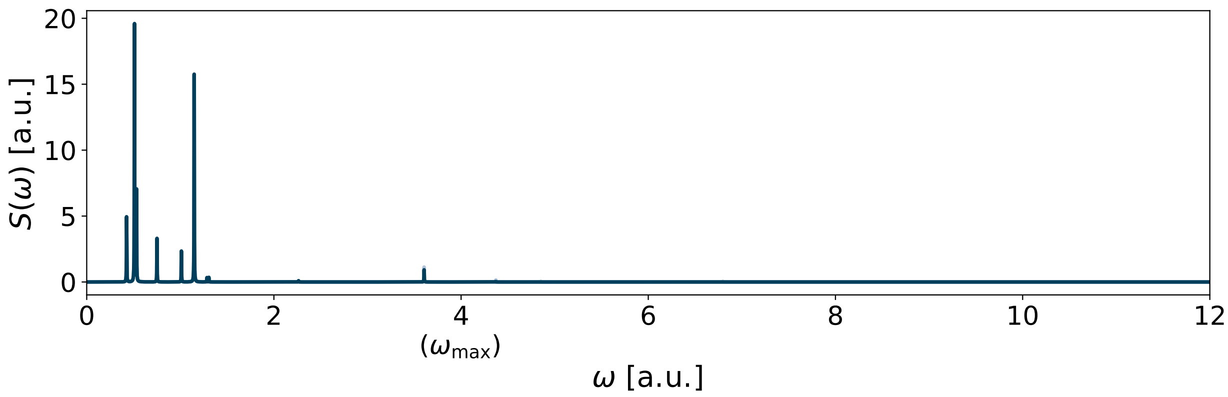

Figure S14: Effects of filtering the absorption spectrum of H_2 using the aug-ucc-pVTZ basis in a RT-TDDFT simulation. The dark line shows the filtered spectrum, while the faded line shows the unfiltered spectrum.

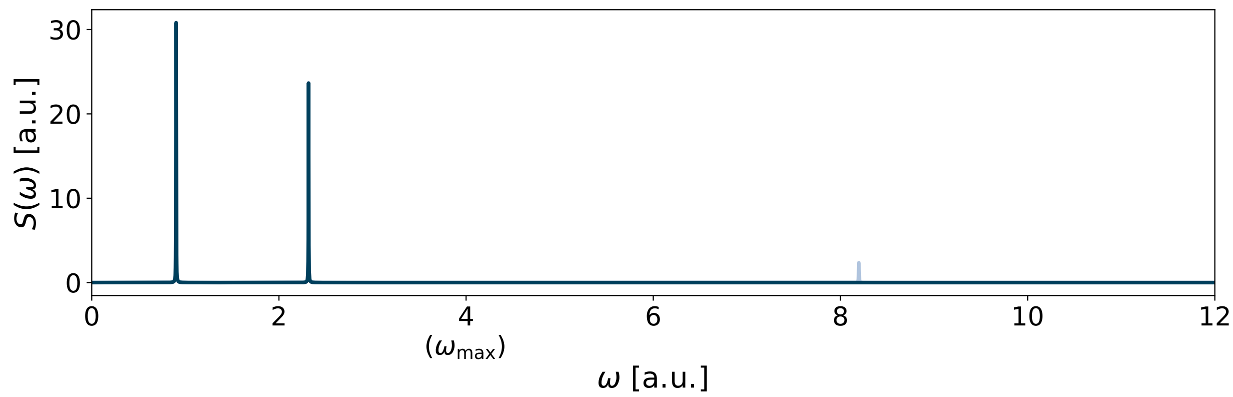

Figure S15: Effects of filtering the absorption spectrum of He using the aug-ucc-pVTZ basis in a RT-TDDFT simulation. The dark line shows the filtered spectrum, while the faded line shows the unfiltered spectrum.

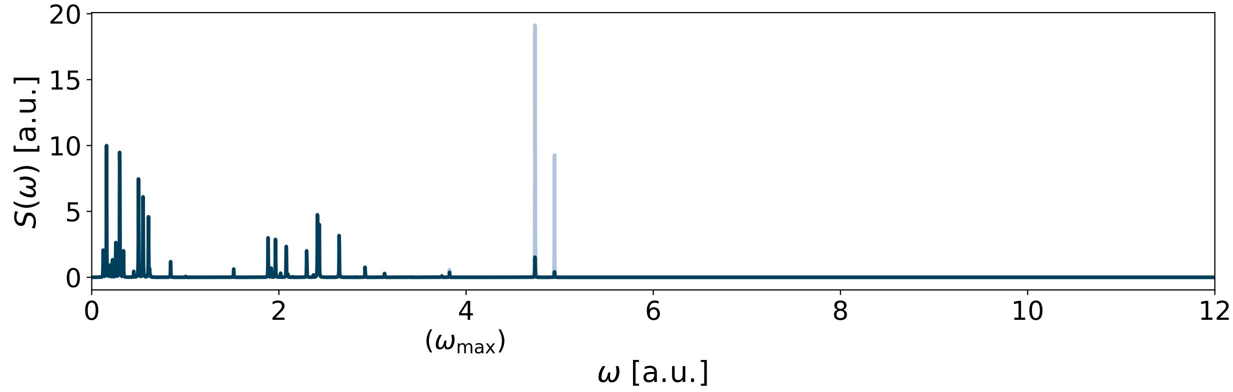

Figure S16: Effects of filtering the absorption spectrum of LiH using the aug-ucc-pVDZ basis in a RT-TDDFT simulation. The dark line shows the filtered spectrum, while the faded line shows the unfiltered spectrum.

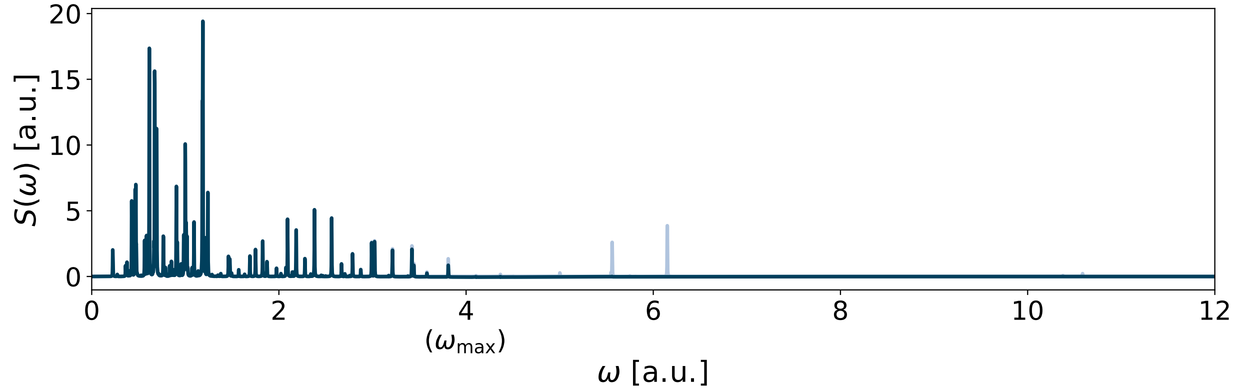

Figure S17: Effects of filtering the absorption spectrum of NH_3 using the aug-ucc-pVDZ basis in a RT-TDDFT simulation. The dark line shows the filtered spectrum, while the faded line shows the unfiltered spectrum.

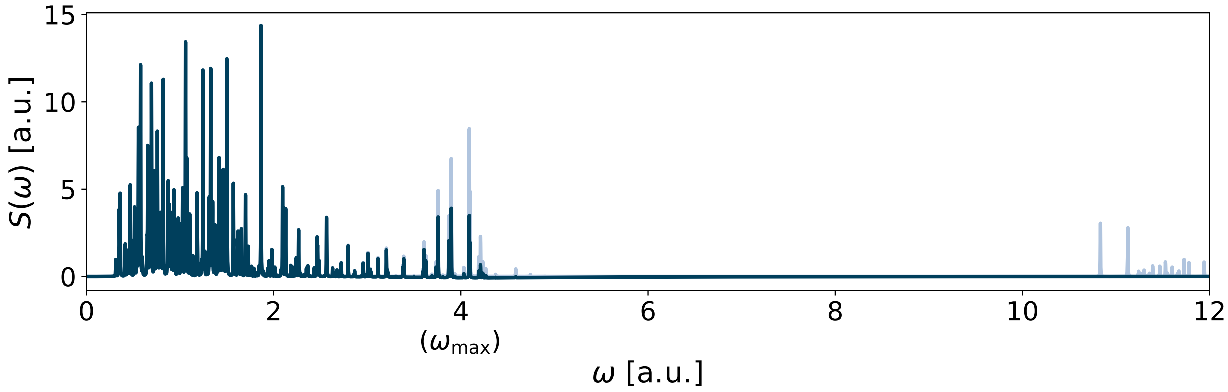

Figure S18: Effects of filtering the absorption spectrum of CH_2O using the aug-cc-pVDZ basis in a RT-TDCIS simulation. The dark line shows the filtered spectrum, while the faded line shows the unfiltered spectrum.

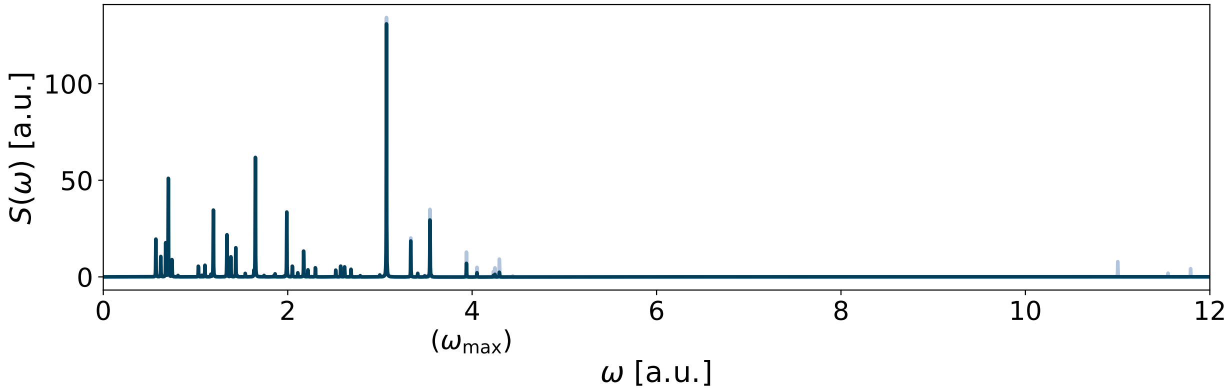

Figure S19: Effects of filtering the absorption spectrum of CO_2 using the cc-pVDZ basis in a RT-TDCIS simulation. The dark line shows the filtered spectrum, while the faded line shows the unfiltered spectrum.

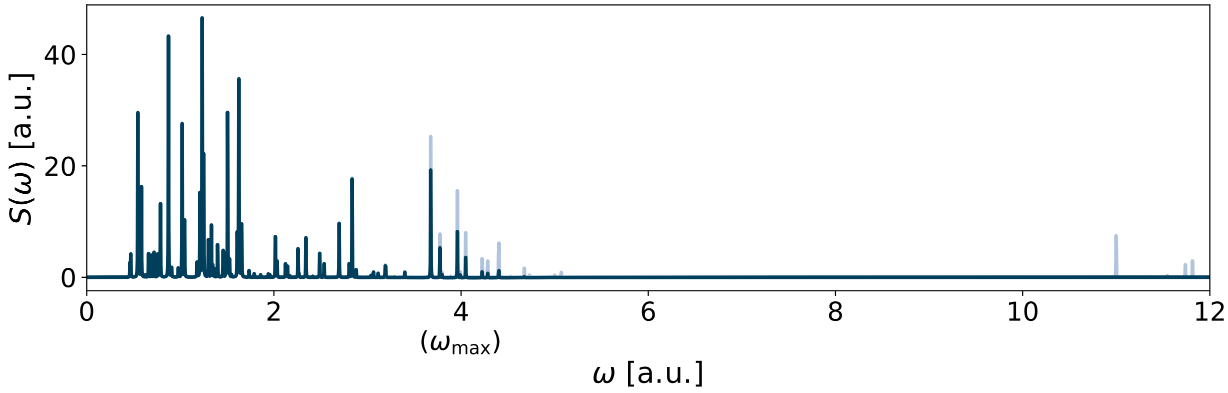

Figure S20: Effects of filtering the absorption spectrum of CO_2 using the aug-cc-pVDZ basis in a RT-TDCIS simulation. The dark line shows the filtered spectrum, while the faded line shows the unfiltered spectrum.

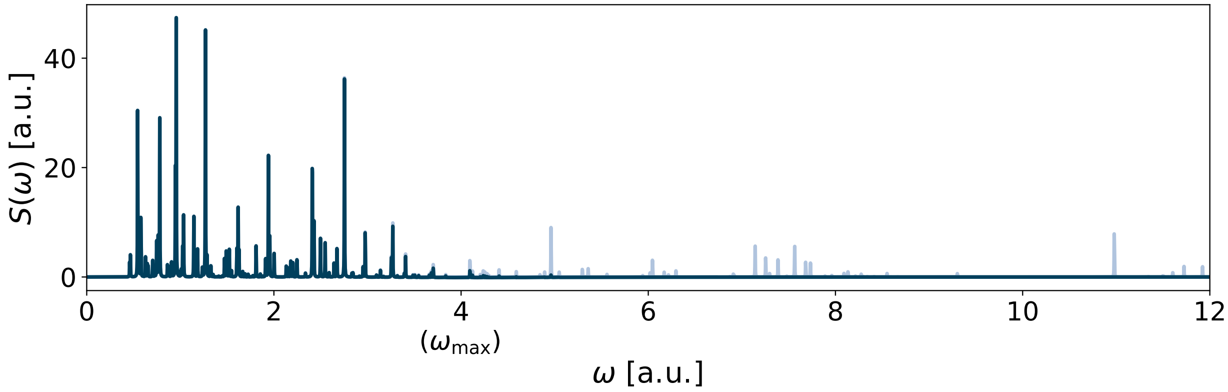

Figure S21: Effects of filtering the absorption spectrum of CO_2 using the aug-cc-pVTZ basis in a RT-TDCIS simulation. The dark line shows the filtered spectrum, while the faded line shows the unfiltered spectrum.

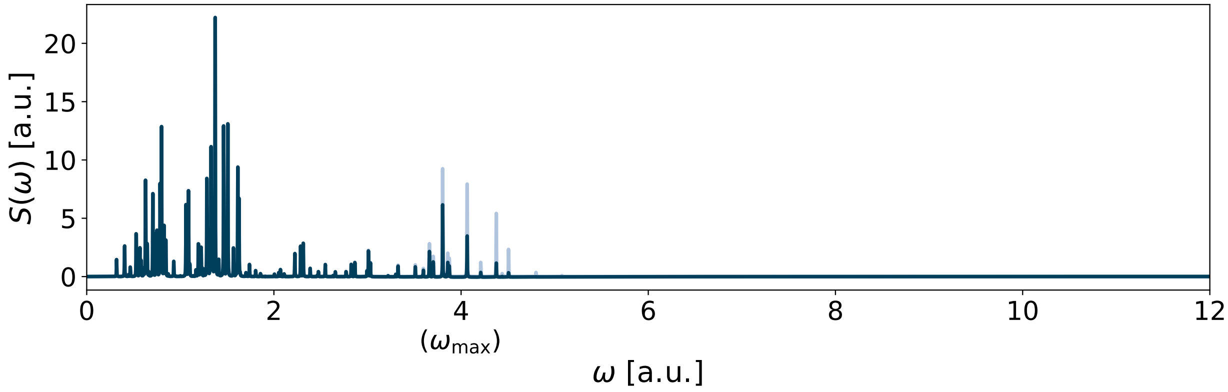

Figure S22: Effects of filtering the absorption spectrum of H_2O using the aug-cc-pVDZ basis in a RT-TDCIS simulation. The dark line shows the filtered spectrum, while the faded line shows the unfiltered spectrum.

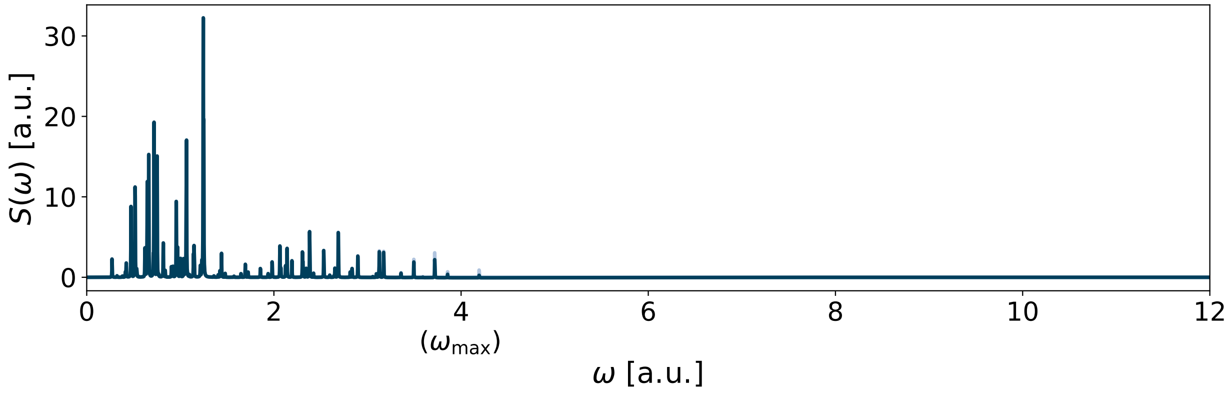

Figure S23: Effects of filtering the absorption spectrum of NH_3 using the aug-cc-pVDZ basis in a RT-TDCIS simulation. The dark line shows the filtered spectrum, while the faded line shows the unfiltered spectrum.

times T^u_{ver} for $u = x, y, z$ are listed in the figures. The maximum trajectory length was set to $T^u_{\text{ver}} = 1000\,\text{a.u.}$, such that spectra with $T^u_{\text{ver}} = 1000\,\text{a.u.}$ in any direction are deemed not converged spectra.

The results of the fitting of the time-dependent dipole moment from RT-TDDFT calculations are shown in Figs. S24 to S27 and S29 to S40, while Figs. S41 to S46 shows the resulting spectra from fitting the dipole moment from RT-TDCIS calculations.

The resulting spectrum from fitting the electric dipole moment using molecular orbital decomposition (dashed orange line) is compared to its reference spectrum (solid dark green line) in Fig. S28. This case uses C_6H_6 with the aug-ucc-pVDZ basis set, from RT-TDDFT calculations.

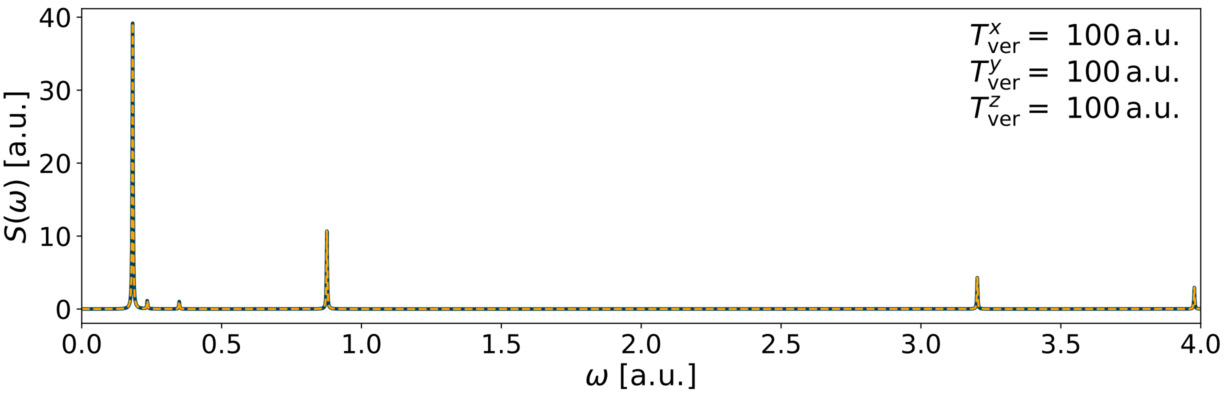

Figure S24: Spectrum of Be using the aug-ucc-pVTZ basis in a RT-TDDFT simulation. The spectrum of the approximated dipole moment is shown by the dashed yellow line, while the solid dark line is the reference spectrum.

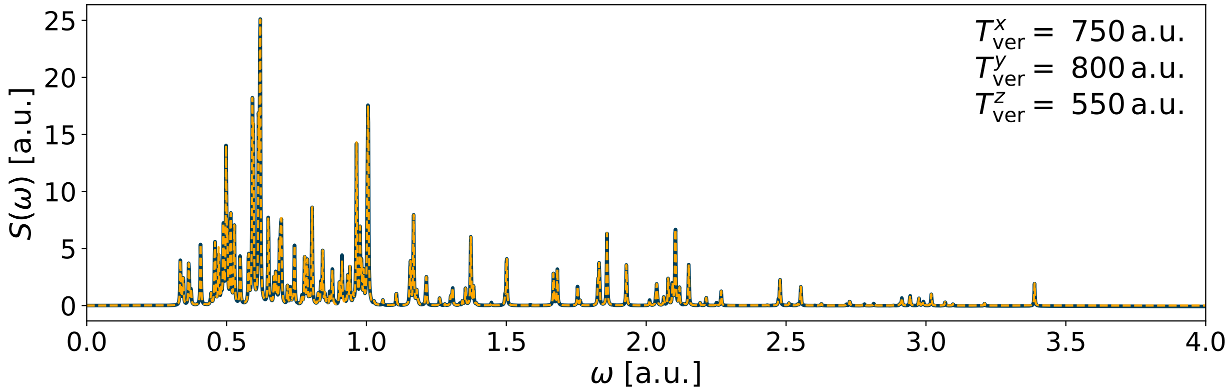

Figure S25: Spectrum of C_2H_6 using the aug-ucc-pVDZ basis in a RT-TDDFT simulation. The spectrum of the approximated dipole moment is shown by the dashed yellow line, while the solid dark line is the reference spectrum.

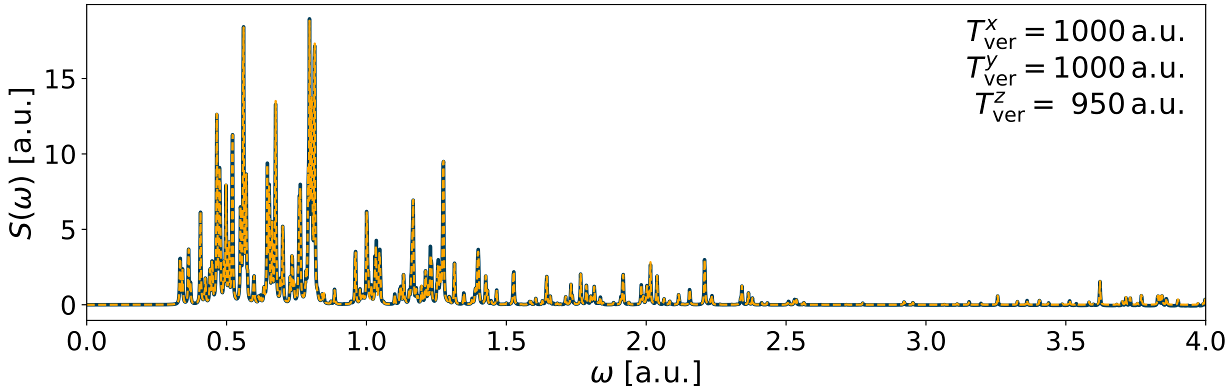

Figure S26: Spectrum of C_2H_6 using the aug-ucc-pVTZ basis in a RT-TDDFT simulation. The spectrum of the approximated dipole moment is shown by the dashed yellow line, while the solid dark line is the reference spectrum.

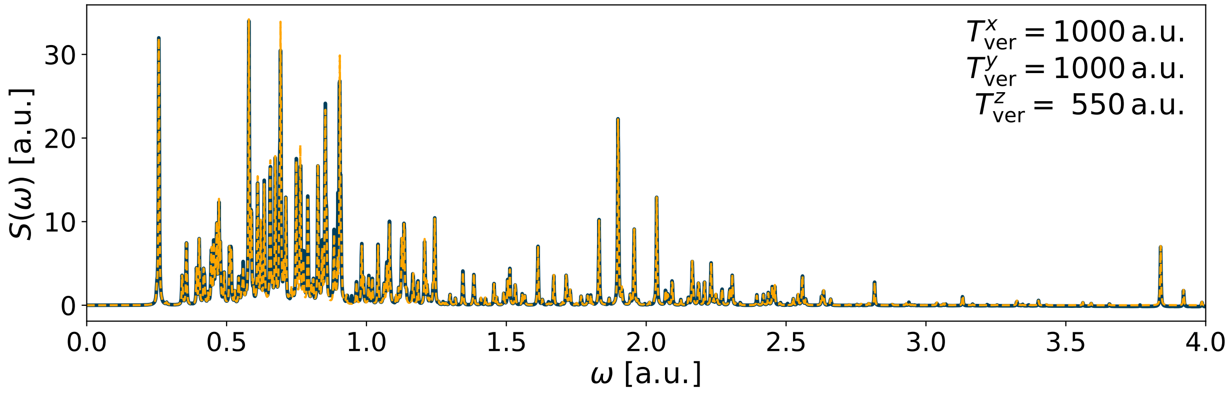

Figure S27: Spectrum of C_6H_6 using the aug-ucc-pVDZ basis in a RT-TDDFT simulation. The spectrum of the approximated dipole moment is shown by the dashed yellow line, while the solid dark line is the reference spectrum.

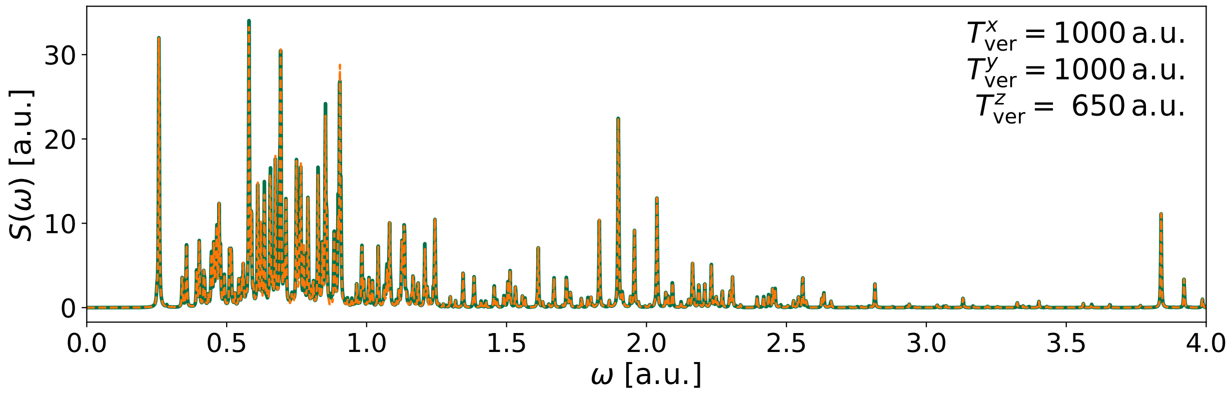

Figure S28: Spectrum of C_6H_6 using the aug-ucc-pVDZ basis in a RT-TDDFT simulation, discarding high-energy molecular orbital components. The spectrum of the approximated dipole moment using molecular orbital decomposition is shown by the dashed orange line, while the solid dark green is the reference spectrum.

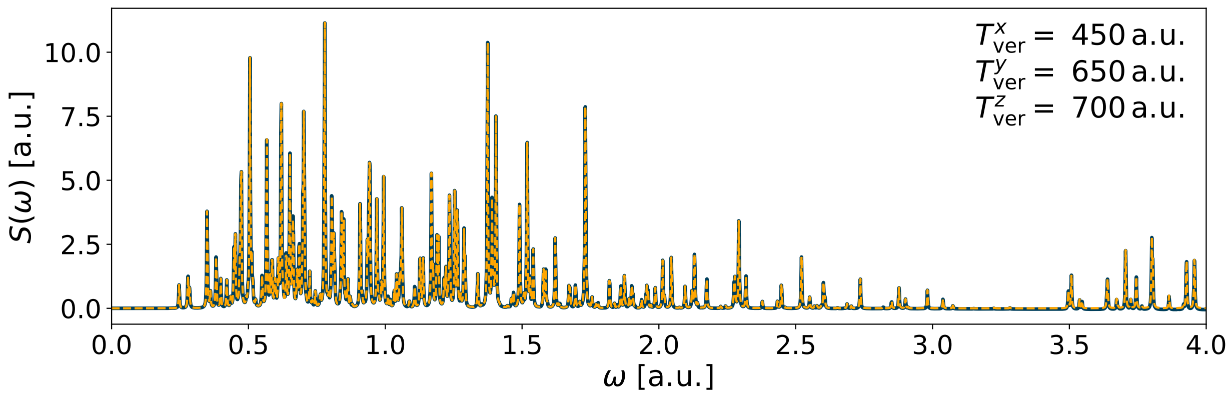

Figure S29: Spectrum of CH_2O using the aug-ucc-pVDZ basis in a RT-TDDFT simulation. The spectrum of the approximated dipole moment is shown by the dashed yellow line, while the solid dark line is the reference spectrum.

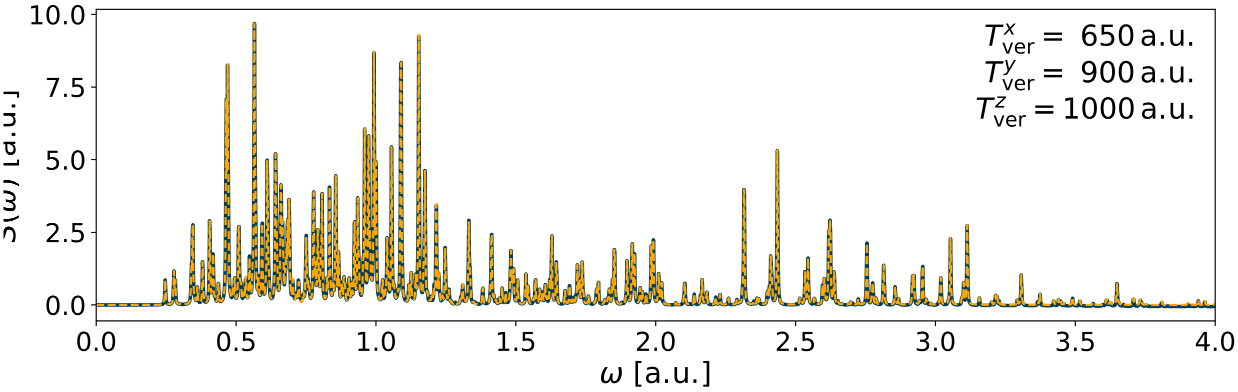

Figure S30: Spectrum of CH_2O using the aug-ucc-pVTZ basis in a RT-TDDFT simulation. The spectrum of the approximated dipole moment is shown by the dashed yellow line, while the solid dark line is the reference spectrum.

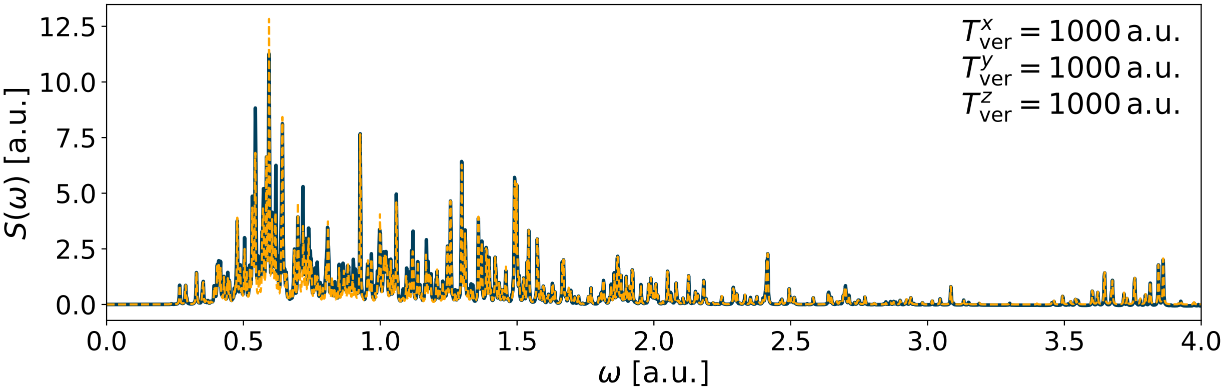

Figure S31: Spectrum of CH_3OH using the aug-ucc-pVDZ basis in a RT-TDDFT simulation. The spectrum of the approximated dipole moment is shown by the dashed yellow line, while the solid dark line is the reference spectrum.

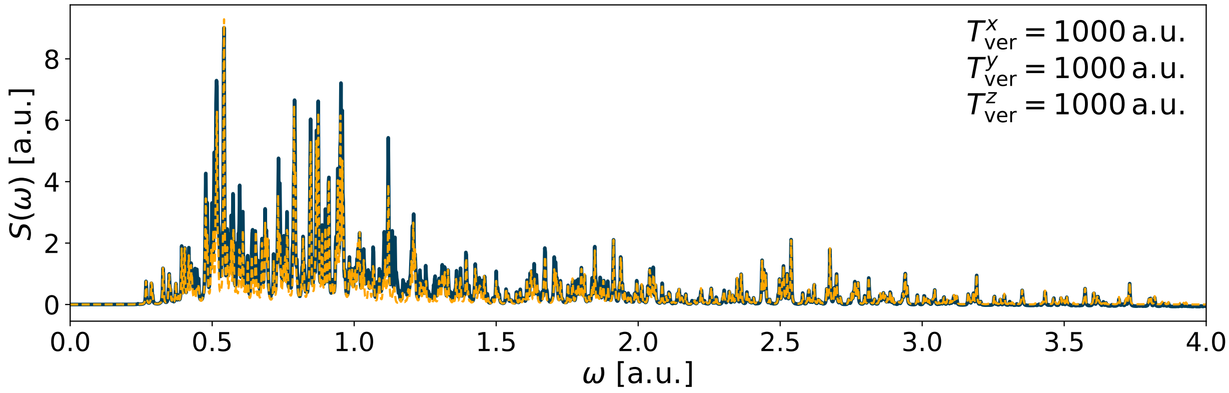

Figure S32: Spectrum of CH_3OH using the aug-ucc-pVTZ basis in a RT-TDDFT simulation. The spectrum of the approximated dipole moment is shown by the dashed yellow line, while the solid dark line is the reference spectrum.

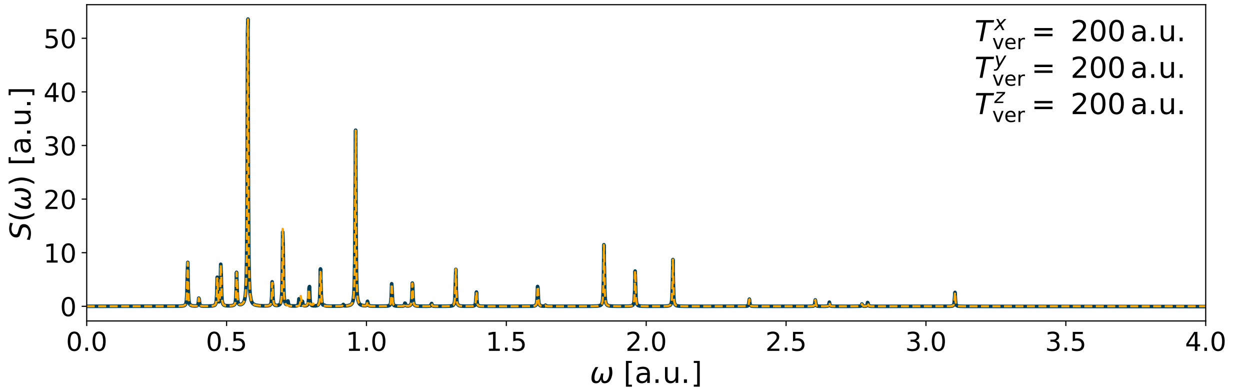

Figure S33: Spectrum of CH_4 using the aug-ucc-pVDZ basis in a RT-TDDFT simulation. The spectrum of the approximated dipole moment is shown by the dashed yellow line, while the solid dark line is the reference spectrum.

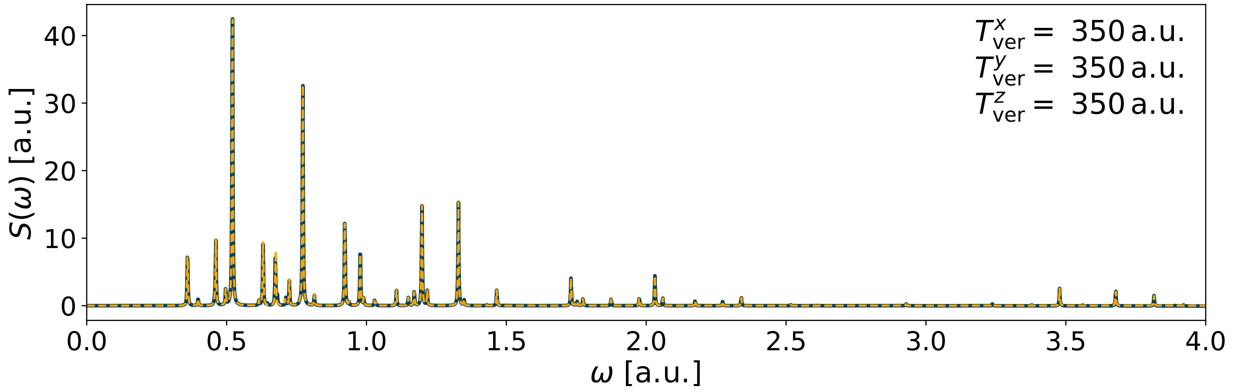

Figure S34: Spectrum of CH_4 using the aug-ucc-pVTZ basis in a RT-TDDFT simulation. The spectrum of the approximated dipole moment is shown by the dashed yellow line, while the solid dark line is the reference spectrum.

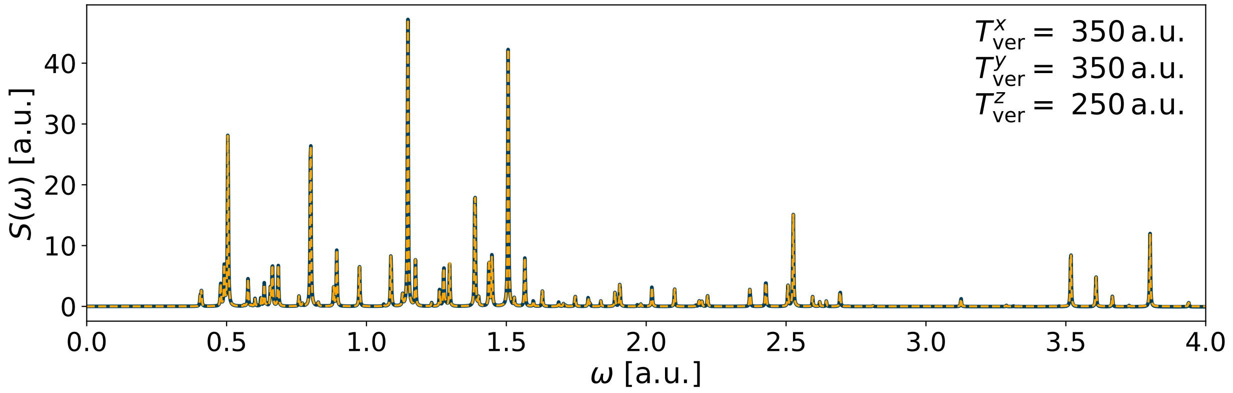

Figure S35: Spectrum of CO_2 using the aug-ucc-pVDZ basis in a RT-TDDFT simulation. The spectrum of the approximated dipole moment is shown by the dashed yellow line, while the solid dark line is the reference spectrum.

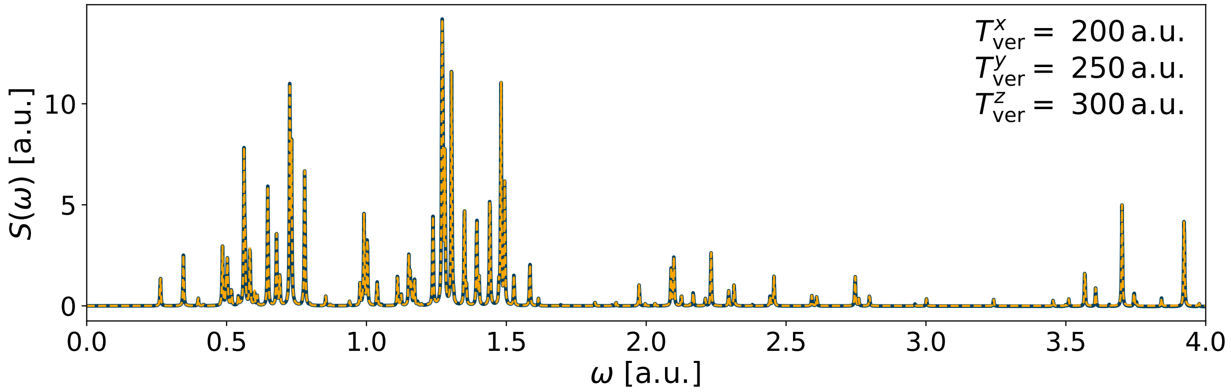

Figure S36: Spectrum of H_2O using the aug-ucc-pVDZ basis in a RT-TDDFT simulation. The spectrum of the approximated dipole moment is shown by the dashed yellow line, while the solid dark line is the reference spectrum.

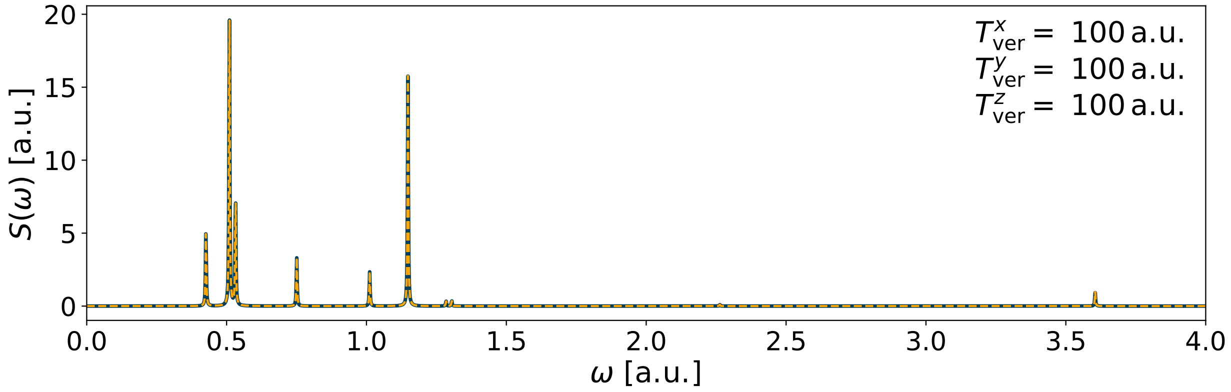

Figure S37: Spectrum of H_2 using the aug-ucc-pVTZ basis in a RT-TDDFT simulation. The spectrum of the approximated dipole moment is shown by the dashed yellow line, while the solid dark line is the reference spectrum.

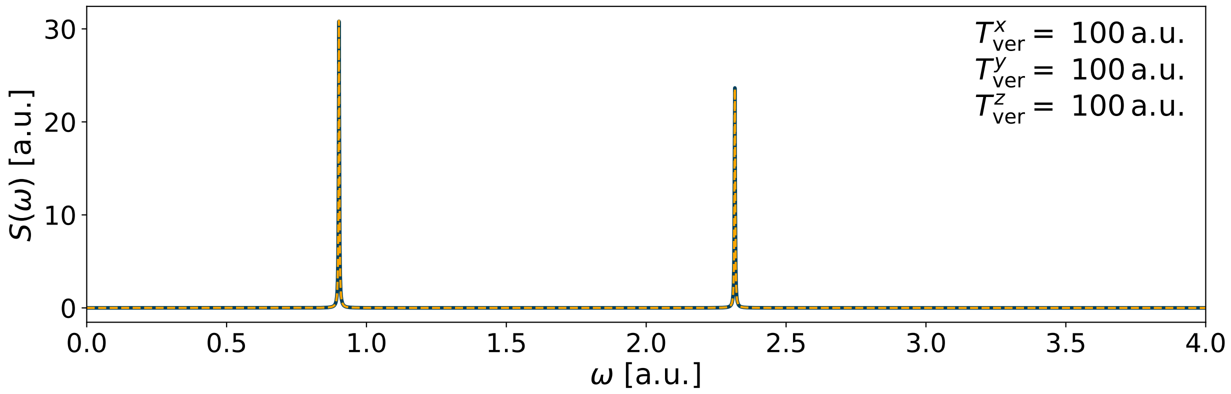

Figure S38: Spectrum of He using the aug-ucc-pVTZ basis in a RT-TDDFT simulation. The spectrum of the approximated dipole moment is shown by the dashed yellow line, while the solid dark line is the reference spectrum.

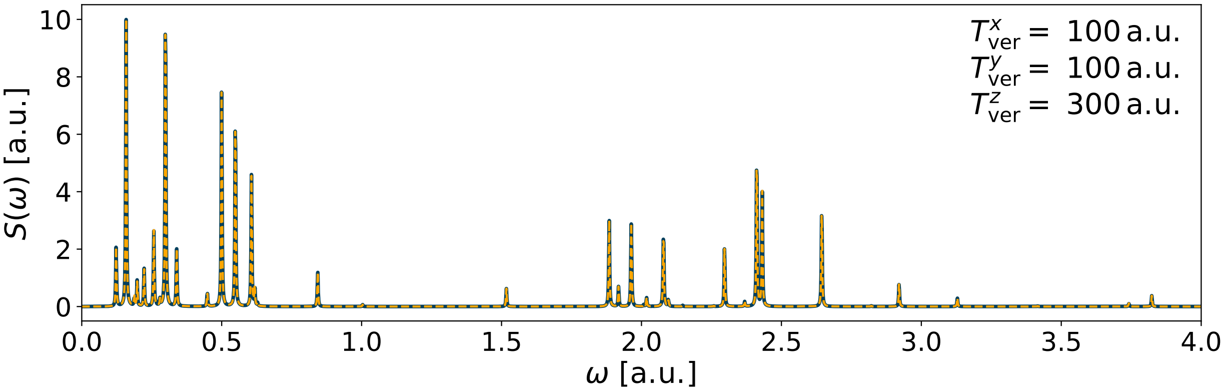

Figure S39: Spectrum of LiH using the aug-ucc-pVDZ basis in a RT-TDDFT simulation. The spectrum of the approximated dipole moment is shown by the dashed yellow line, while the solid dark line is the reference spectrum.

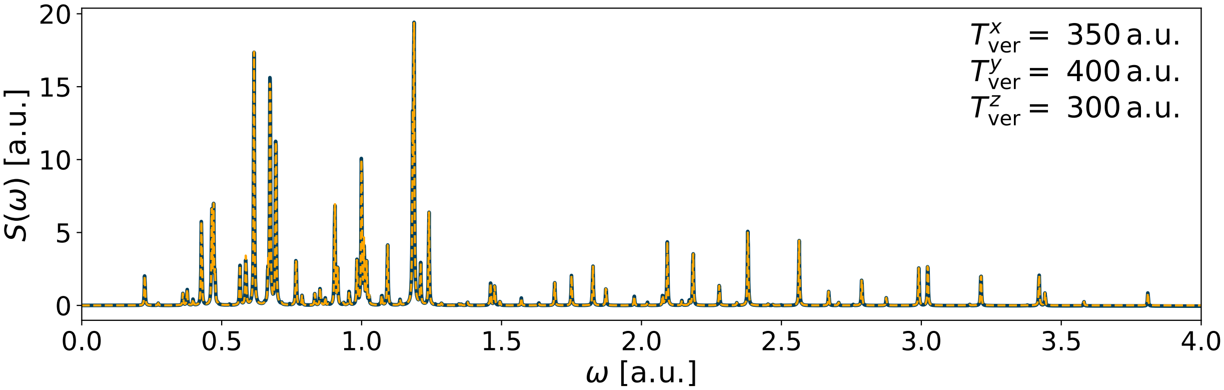

Figure S40: Spectrum of NH_3 using the aug-ucc-pVDZ basis in a RT-TDDFT simulation. The spectrum of the approximated dipole moment is shown by the dashed yellow line, while the solid dark line is the reference spectrum.

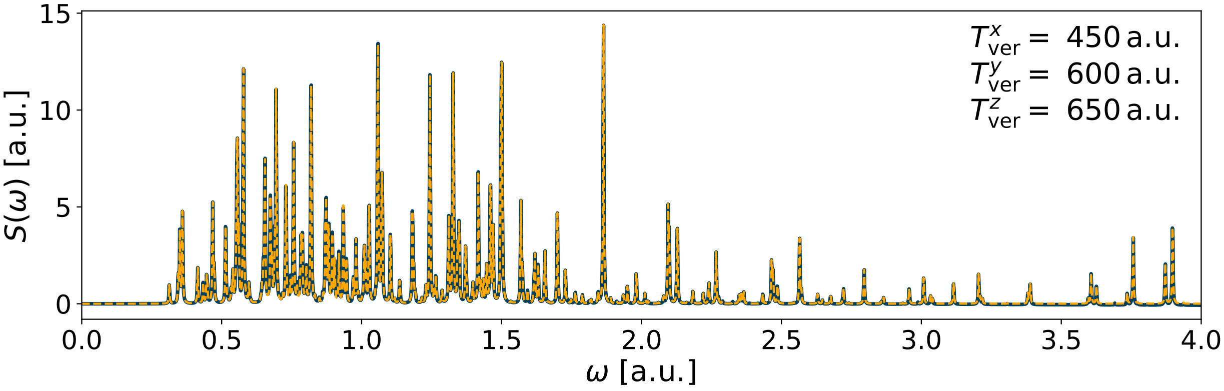

Figure S41: Spectrum of CH_2O using the aug-cc-pVDZ basis in a RT-TDCIS simulation. The spectrum of the approximated dipole moment is shown by the dashed yellow line, while the solid dark line is the reference spectrum.

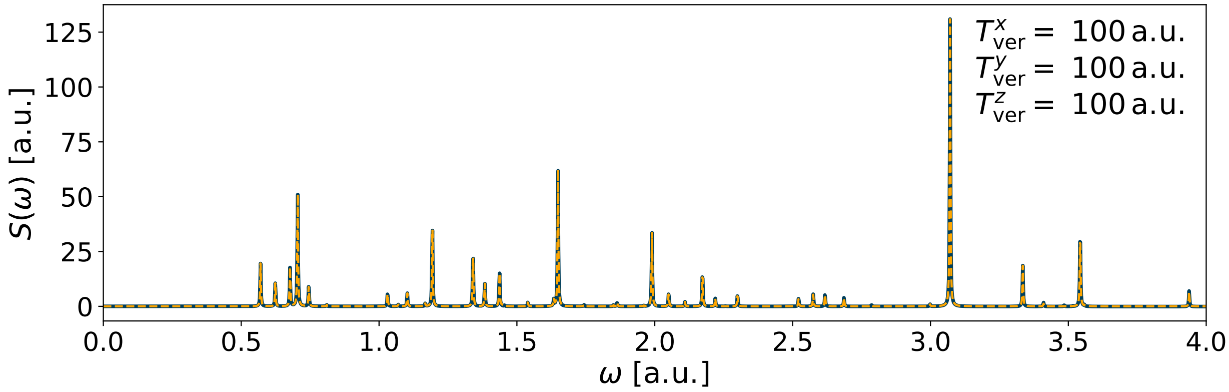

Figure S42: Spectrum of CO_2 using the cc-pVDZ basis in a RT-TDCIS simulation. The spectrum of the approximated dipole moment is shown by the dashed yellow line, while the solid dark line is the reference spectrum.

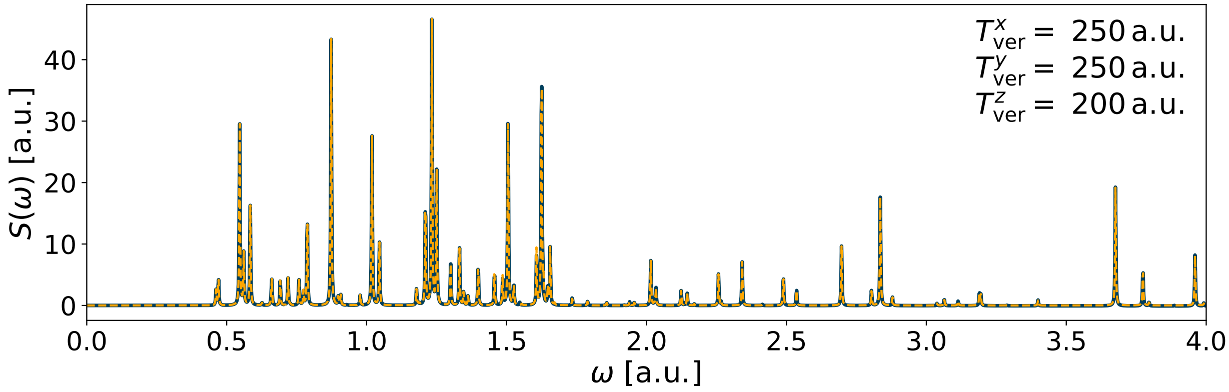

Figure S43: Spectrum of CO_2 using the aug-cc-pVDZ basis in a RT-TDCIS simulation. The spectrum of the approximated dipole moment is shown by the dashed yellow line, while the solid dark line is the reference spectrum.

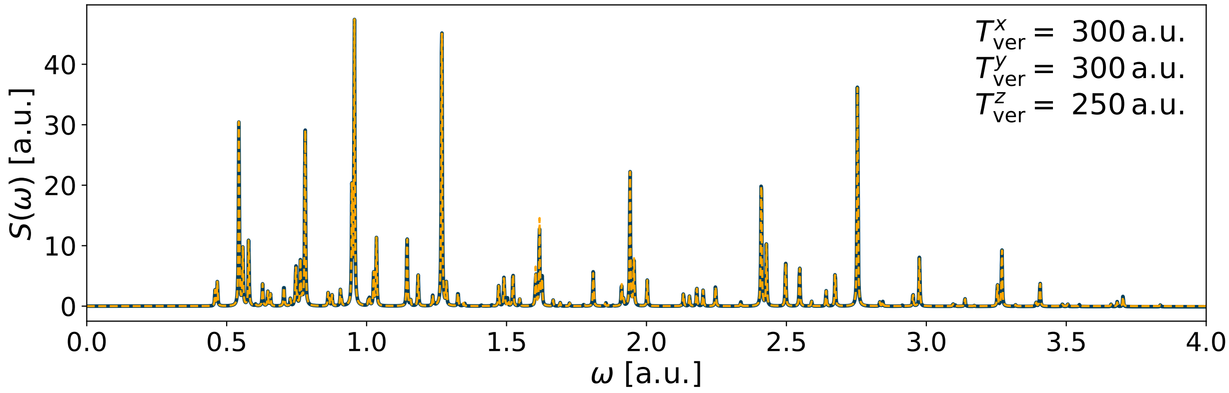

Figure S44: Spectrum of CO_2 using the aug-cc-pVTZ basis in a RT-TDCIS simulation. The spectrum of the approximated dipole moment is shown by the dashed yellow line, while the solid dark line is the reference spectrum.

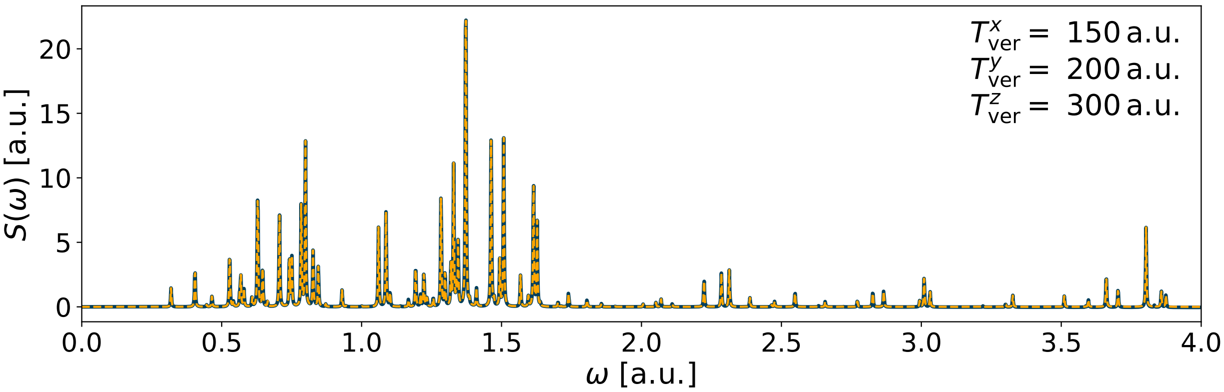

Figure S45: Spectrum of H_2O using the aug-cc-pVDZ basis in a RT-TDCIS simulation. The spectrum of the approximated dipole moment is shown by the dashed yellow line, while the solid dark line is the reference spectrum.

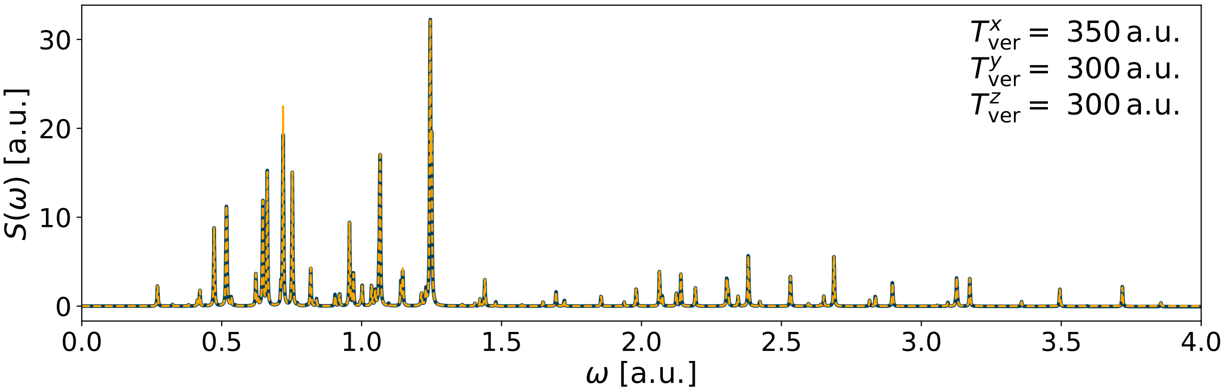

Figure S46: Spectrum of NH_3 using the aug-cc-pVDZ basis in a RT-TDCIS simulation. The spectrum of the approximated dipole moment is shown by the dashed yellow line, while the solid dark line is the reference spectrum.
